# Age-dependent neuroinflammation and cognitive decline in a novel Ala152Thr-Tau transgenic mouse model of PSP and AD

**DOI:** 10.1186/s40478-016-0281-z

**Published:** 2016-02-25

**Authors:** Astrid Sydow, Katja Hochgräfe, Stefanie Könen, Daniela Cadinu, Dorthe Matenia, Olga Petrova, Maria Joseph, Frank Johannes Dennissen, Eva-Maria Mandelkow

**Affiliations:** German Center for Neurodegenerative Diseases (DZNE), Ludwig-Erhard-Allee 2, 53175 Bonn, Germany; Max-Planck-Institute for Metabolism Research, Hamburg Outstation, c/o DESY, Notkestrasse 85, 22607 Hamburg, Germany; CAESAR Research Center, Ludwig-Erhard-Allee 2, 53175 Bonn, Germany

**Keywords:** Progressive supranuclear palsy, Tau mutation, Mouse model, Inflammation, Autophagy, Behavior

## Abstract

**Introduction:**

Mutations of Tau are associated with several neurodegenerative disorders. Recently, the Tau mutation A152T was described as a novel risk factor for frontotemporal dementia spectrum disorders and Alzheimer disease. In vitro Tau-A152T shows a decreased binding to microtubules and a reduced tendency to form abnormal fibers.

**Results:**

To study the effects of this mutation we generated a mouse model expressing human full-length Tau with this mutation (hTau40^AT^). At young age (2–3 months) immunohistological analysis reveals pathological Tau conformation and Tau-hyperphosphorylation combined with Tau missorting into the somatodendritic compartment of neurons. With increasing age there is Tau aggregation including co-aggregates of endogenous mouse Tau and exogenous human Tau, accompanied by loss of synapses (especially presynaptic failure) and neurons. From ~10 months onwards the mice show a prominent neuroinflammatory response as judged by activation of microglia and astrocytes. This progressive neuroinflammation becomes visible by in vivo bioluminescence imaging after crossbreeding of hTau40^AT^ mice and Gfap-luciferase reporter mice. In contrast to other Tau-transgenic models and Alzheimer disease patients with reduced protein clearance, hTau40^AT^ mice show a strong induction of autophagy. Although Tau-hyperphosphorylation and aggregation is also present in spinal cord and motor cortex (due to the Thy1.2 promoter), neuromotor performance is not affected. Deficits in spatial reference memory are manifest at ~16 months and are accompanied by neuronal death.

**Conclusions:**

The hTau40^AT^ mice mimic pathological hallmarks of tauopathies including a cognitive phenotype combined with pronounced neuroinflammation visible by bioluminescence. Thus the mice are suitable for mechanistic studies of Tau induced toxicity and in vivo validation of neuroprotective compounds.

**Electronic supplementary material:**

The online version of this article (doi:10.1186/s40478-016-0281-z) contains supplementary material, which is available to authorized users.

## Introduction

Tauopathies define a class of neurodegenerative diseases associated with the pathological deposition of Tau protein in different substructures of the human brain. The neuropathological profiles and clinical symptoms are rather heterogeneous and dependent on the affected brain areas [54]. The most prominent tauopathy is Alzheimer disease (AD), where insoluble aggregated Tau fibers ("PHFs") are the major component of neurofibrillary lesions. The pathology propagates from transentorhinal regions to the limbic system and neocortical areas as described by the Braak stages for AD-patients and transgenic mice [15, 22, 41, 63]. As a consequence of synapse loss and neuronal death in the affected brain areas, AD is clinically characterized by short- and long-term memory loss [103]. Other tauopathies include the group of frontotemporal dementias (FTD) with the major pathological subtypes of progressive supranuclear palsy (PSP), corticobasal degeneration (CBD), Pick disease (PiD), argyrophilic grain disease (AgD), multiple system tauopathy with dementia (MSTD) and frontotemporal dementia and parkinsonism linked to chromosome 17 (FTDP-17) [79]. Major risk factors for FTDs are mutations within the microtubule associated protein Tau (*MAPT)* gene located on chromosome 17 (17q21) [4]. Most *MAPT* mutations are clustered in exons 9–13, encoding for the Tau repeat domain and flanking regions responsible for microtubule (MT) binding. Consequently, these Tau mutations destabilize microtubules and enhance Tau-aggregation since the β-sheet rich repeat domain plays a major role in Tau filament assembly [70].

Recently, a rare *MAPT* p.A152T mutation was identified as a novel risk factor among patients diagnosed with PSP, AD, PD, CBD and unclassifiable tauopathy presenting with atypical clinical and neuropathological features [20, 38, 55, 57, 60]. Besides p.A152T, several other *MAPT* mutations cause clinical and neuropathological phenotypes resembling PSP, i.e. R5L, N279K, L284R, homozygous ΔN296, G303V, S305S, S352L and R406W, and an extended H1 haplotype [8, 90, 113].

The *MAPT* p.A152T mutation is located in exon 7 encoding the N-terminal part or “projection domain” of Tau, which is far from MT binding domain [57]. In comparison to wild-type Tau, hTau40^AT^ is less efficient in stabilizing MT, it reduces the aggregation into filaments and enhances oligomeric structures in vitro [20]. Expression of hTau40^AT^ in human induced pluripotent stem cells (hIPSC) shows an increased Tau- fragmentation and phosphorylation leading to axonal degeneration [32]. However, it is still not known how the mutant hTau40^AT^ contributes to neurotoxicity.

To this end we generated a new mouse model expressing human full-length Tau (hTau40, 2N4R) with the point mutation A152T (hTau40^AT^ for short) and characterized the pathological and functional effects under physiological conditions. The transgenic hTau40^AT^ mice develop a progressive Tau pathology including Tau conformational changes, Tau-hyperphosphorylation and Tau-aggregation. This is accompanied by loss of synapses (especially presynaptic failure), neuronal death and upregulation of protein clearance mechanisms such as autophagy. In addition the expression of hTau40^AT^ causes a marked increase of astrocytic and microglia activity, indicating a strong neuroinflammatory response. In spite of pan-neuronal expression in the brain and spinal cord, hTau40^AT^ mice exhibit intact motor functions but develop cognitive decline at advanced age (~16 mo). The study shows that hTau40^AT^ -expression at low near-physiological levels (1-2-fold over endogenous Tau) is sufficient to induce a severe neuropathology leading to functional deficits and neurodegeneration in vivo, consistent with a neurotoxic gain of function. Thus the new tauopathy mouse model expressing hTau40^AT^ is suitable for mechanistic studies of Tau induced toxicity and for in vivo validation of neuroprotective compounds.

## Materials and methods

### Generation of hTau40^AT^ mice

To achieve expression at moderate levels the transgene (human full-length Tau carrying the mutation A152T) was inserted into the ROSA26-locus [33] of C57BL/6 N embryonic stem (ES) cells and injected into BALB/c blastocysts (Taconic). Injected blastocysts were transferred into the uterine horn of pseudopregnant NMR1 females. Highly chimeric mice were bred to C57BL/6 N females. Germline transmission was identified by the presence of black offspring. The transgene expression is controlled by the neuron specific murine Thy1.2 promoter and occurs pan-neuronally in brain and spinal cord. The present study shows data of heterozygous hTau40^AT^ mice with identical C57BL/6 N background. Non-transgenic littermates were used as negative controls. All animals were housed and tested according to standards of the German Animal Welfare Act. hTau40^AT^ mice were identified by PCR using primers 5’-AGCACCCTTAGTGGATGAGG-3’ and 5’-TTGTCATCGCTTCCAGTCC-3’, amplifying the human Tau target fragment.

### Biochemical analysis

Sarcosyl extraction, total protein preparation and western blots analysis were performed as described [75]. Briefly, 3–40 μg of total protein or 3 μl of sarcosyl extraction lysates from tissues (cortex, hippocampus, cerebellum, spinal cord) were separated on 10 % SDS-gels or gradient gels (4 %–20 %, Biorad) and transferred to PVDF-membranes for detection with the following antibodies: K9JA (1:20000, Dako), 12E8 (pS262/pS356, 1:1000, a gift from Dr. P. Seubert, Elan Pharma, South San Francisco, CA), AT8 (pSer202/pThr205, 1:500, Thermo Scientific), PHF-1 (pS396/pS404, 1:50, a gift from Dr. P. Davies, Albert Einstein College of Medicine, NY), synaptophysin (1:20000, Sigma), PSD95 (1:2000, Dianova), NeuN (1:1000, Millipore), PSMD13 (1:1000, Abcam), Prot 20S C2 (1:1000, Abcam), LC3 (1:1000, Novus Biologicals), p62 (1:2500, Abnova), Iba1 (1:1000, WAKO), iNOS (1:1000, Abcam), CD11b (1:500, Novus) and GFAP (1:2000, Sigma). Blots were normalized by the concentration of ß-actin (1:20000, Sigma) or GAPDH (1:10000, Sigma), visualized with ECL Plus detection system (GE Healthcare) and analyzed by densitometry (LAS 3000/ChemiBis, AIDA software). Bars represent mean ± SEM; *n* = 3–17.

### Histological analysis

Immunohistochemistry was performed with 5 μm paraffin sections as described [75]. Tissue (brain and spinal cord) was fixed in histofix (Roth; 4 % PFA, pH 7.4 for 24 h) and dehydrated by a series of ethanol and chloroform. Sagittal brain and coronal spinal cord sections were incubated with primary antibodies prepared in 1 % horse serum overnight at 4 °C. The following antibodies were used: TauY9 (1:2000, Enzo), 12E8 (pS262/pS356, 1:2000) (a gift from Dr. P. Seubert, Elan Pharma, South San Francisco, CA), AT180 (pThr231/pSer235, 1:500, Pierce), AT8 (pSer202/pThr205, 1:500, Thermo Scientific), Alz-50 (1:50) and PHF-1 (pS396/pS404, 1:50) (Alz-50 and PHF-1 are gifts from Dr. P. Davies, Albert Einstein College of Medicine, NY), Iba1 (1:1000, Wako), GFAP (1:2000, Sigma), S100b (1:100000, Novus), IL1ß (1:25, SantaCruz), TLR2 (1:150, Millipore). Inflammatory markers like CD11b (1:5000, Serotec) and CD45 (1:500, Serotec) were analyzed using 20 μm sagittal cryo sections. Counterstaining with hematoxylin (Roth) was performed according to the company´s instructions. To visualize co-pathologies (neuroinflammation vs. Tau phosphorylation), sections were first incubated with antibodies against inflammatory markers and developed with Vectastain Universal Elite ABC kit + DAB (Vector Laboratories). Afterwards sections were re-incubated with primary antibodies against Tau-phospho epitopes and developed with Alkaline Phosphatase Universal kit + Alkaline Phosphatase substrate kit III (Vector Laboratories). Stainings were performed on paraffin sections of 4–6 mice, respectively, for genotype, age and antibody used.

### Fluoro Jade C staining

To label neurodegeneration, 30 μm floating sections of 3 WT and 3 hTau40^AT^ mice were mounted on gelatin coated slides and rehydrated in a series of ethanol. Tissue autofluorescence was reduced by 0.06 % potassium permanganate treatment for 15 min. Washed slices were stored for 30 min in 0.001 % Fluoro Jade C staining solution (Merck) in the dark. Rinsed and dried slides were immersed in xylene and coverslipped with Histokitt (Roth). Fluoro Jade C staining was analyzed using FITC filter settings.

### Gallyas silver staining

5 μm paraffin sections were stained as published [15]. To visualize co-pathologies (neuroinflammation vs. Tau-aggregation), Gallyas silver stained sections were incubated with antibodies against inflammatory markers and developed with Vectastain Universal Elite ABC kit + DAB (Vector Laboratories). Stainings were performed on paraffin sections of 4 mice, respectively for genotype and age.

### Thioflavin S staining

Autofluorescence of 5 μm paraffin brain sections was quenched [99], sections were incubated in 0.05 % Thioflavin S (Sigma) for 8 min, and excess Thioflavin S was removed by brief washing with 80%EtOH and three washing steps in large volumes of tap water. Stained sections were stored in cold 3xPBS for 30 min to avoid photobleaching. After rinsing in 1xPBS, the tissue was counterstained with TOPRO-3 (nuclear marker; Invitrogen Molecular Probes) and mounted in Aqua Poly/Mount (Polysciences Inc.). Analysis was performed on paraffin sections of 4 mice, respectively, for genotype and age.

### Golgi-staining and quantification of spines

For Golgi-Cox impregnation of neurons [35], the FD rapid GolgiStain TM kit (FD NeuroTechnologies) was used according to the manufacturer’s protocol. 80 μm floating sections of transgenic and WT mice at 10 and 20 months of age were Golgi-impregnated and hippocampal pyramidal CA1- or CA3-neurons were used for quantification of dendritic spines as described [88]. For each mouse (*n* = 2–3 per group), ~10 neurons and 1–2 secondary dendrites per neuron of 20–30 μm lengths were quantified using ImageJ software (NIH). Bars represent mean ± SEM.

### In vivo bioluminescence imaging (BLI) of astrocyte activation

hTau40^AT/C57BL/6N^ mice were crossbred to Tg(Gfap-luc^FVB/N^) reporter mice, expressing firefly luciferase under control of the murine Gfap-promoter [117]. Heterozygous, bigenic Tg(Gfap-luc: hTau40^AT/mixed bkg^) offspring (*n* = 18) were used to monitor luciferase activity as surrogate marker for astrocyte activation and Tau pathology. Heterozygous Tg(Gfap-luc^mixed bkg^) (*n* = 9) were used as controls. Mice of both genders were imaged monthly starting at 3 months of age and continued until 18 months of age.

*In vivo* BLI was performed using an Ivis Lumina II system (Caliper Life Science) according to a standardized protocol. Ten minutes prior to each imaging session, mice received an intraperitoneal (i.p.) injection of 150 mg/kg D-luciferin (Caliper Life Science) dissolved in sterile PBS and the heads of the animals were shaved. Mice were anesthetized using 2 % isoflurane (Abbott) vaporized in a constant O_2_ flow. Anesthesia was maintained during the whole imaging session. Mice were placed into the heated, light-tight imaging chamber of the Ivis Lumina II and the ears were covered using black paper to shield unspecific luminescence signals. A sequence of 6 images taken in 2 min intervals starting at 10 min post i.p. injection was recorded using a highly sensitive charged coupled device camera. Images were analyzed using Living Image 4.0 software (Caliper Life Science). The bioluminescence emission was normalized and the surface radiance was displayed in photons per second per centimeter squared per steradian (photons/s/cm^2^/sr). For quantification of bioluminescence signals, a region of interest (ROI) was defined to convert surface radiance (photons/s/cm^2^/sr) into total flux of the bioluminescent source (photons/s). To compare bioluminescence signals of Tg(Gfap-luc: hTau40^AT/mixed bkg^) and controls, total flux values of each experimental cohort were converted to percentage. Data represent mean values ± standard error of the mean (SEM). Statistical comparisons were accomplished by two-way repeated ANOVA followed by a post hoc Bonferroni´s multiple comparison test using Prism 5.0 (GraphPad Software). The accepted level of significance was *p* < 0.05. Asterisks indicate significant differences between Tg(Gfap-luc: hTau40^AT/mixed bkg^) and controls for each time point (*:*p* < 0.05, **:*p* < 0.01, ***:*p* < 0.001, ****:*p* < 0.0001).

### Housing conditions

Mice were housed in groups of 2–5 animals under standard conditions (23 °C, 40 %–50 % humidity, food and water ad libitum) with a 12 h light/dark cycle (with light on from 7 a.m. to 7 p.m.). After 2 weeks of acclimatization and handling, behavior tests were carried out between 9 a.m. and 4 p.m. Transgenic hTau40^AT^ mice (mixed genders) were tested at 10 months (*n* = 18) and 16 months of age (*n* = 20) and compared to age-matched wild-type control littermates (mixed genders, *n* = 14 and *n* = 11 respectively).

### Morris water maze (MWM)

#### MWM pretraining

A 2 day pretraining protocol was conducted to habituate the mice to swimming and climbing onto a hidden platform (water temperature: 22 °C, 4 trials/day, maximum duration/trial 60s, 60 min inter-trial interval). To avoid any interference with the MWM learning, the pretraining was performed in a different apparatus (Makrolon cage type III, 42 × 26.5 × 15.5 cm) than used for the MWM (circular pool, diameter of 150 cm). The position of the pretraining platform (diameter of 10 cm, 1 cm below the water surface) was randomized and could not be located by orientation via landmarks, thus mice had to swim at random to escape from the water.

#### MWM acquisition and probe trials

Spatial memory abilities were examined in the standard hidden-platform acquisition and retention version of the Morris water maze [77]. A 150 cm circular pool was filled with water opacified with non-toxic white paint (Biofa Primasol 3011), and kept at 22 °C. Four positions around the edge of the tank were arbitrarily designated 1, 2, 3 and 4 thus dividing the tank into four quadrants: target (T), right adjacent (R), opposite (O), and left adjacent (L). A 15 cm round platform was hidden 1 cm beneath the surface of the water at a fixed position in the center of target quadrant. The water maze was equipped with inner maze cues arranged in an asymmetrical manner to facilitate orientation. Each mouse performed 4 swimming trials per day (maximum duration 60s, 10 min inter-trial interval) for five consecutive days. Mice were started from 4 symmetrical positions in a pseudo-randomized order across trials. Mice that failed to find the submerged platform within 60s were guided to the platform, where they remained for 15 s before being returned to their home cage. The time required to locate the hidden escape platform (escape latency), the distance travelled (path length), and swimming speed (velocity) were determined. On acquisition day 3, 4, 5, as well as 2 days after the acquisition phase ended (day 8), a probe trial was conducted with the platform removed and the search pattern of the mice was recorded for 60s. On day 3, 4 and 5 the probe trial was performed between learning trial 2 and 3. The following learning trials 3–4 were carried out with the platform returned to former position inside the target quadrant to avoid extinction. During acquisition and probe trials the Viewer II video tracking system was used to record and analyze behavior (Noldus).

Statistical comparisons between groups were accomplished by two-way repeated ANOVA followed by a post hoc Fisher LSD multiple comparison test. Stars presented in graphs indicate differences between hTau40^AT^ mice and wild-type littermates (MWM acquisition). For analysis of probe trials a two-tailed one sample t-test against chance level (25 %) or a one-way ANOVA with post hoc Newman-Keuls multiple comparison test was done. All data are presented as group mean values with standard error of mean (SEM), the accepted level of significance was *p* < 0.05. Statistical comparisons were performed using STATISTICA 10.0 software (StatSoft Germany), graphs were designed using Prism 5.0 (GraphPad Software). *:*p* < 0.05, **:*p* < 0.01, ***:*p* < 0.001.

## Results

### Generation of hTau40^AT^ mice

**Fig. 1:**
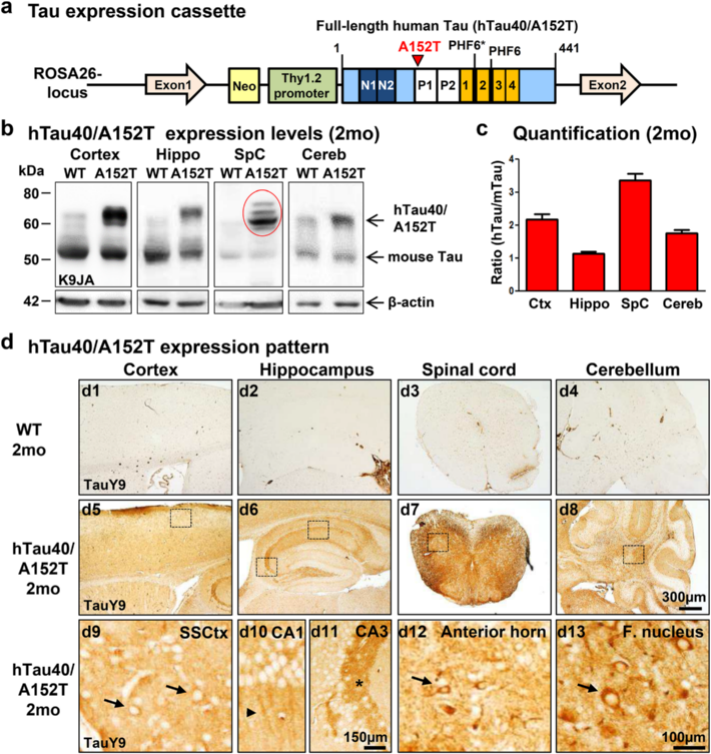
Generation of human hTau40^AT^ mice with the Tau mutation A152T. **a** Illustration of the mutated hTau40^AT^ -gene located in the ROSA26-locus. Scheme represents human full-length Tau (hTau40) with highlighted A152T mutation (*red*), proline-rich domain (*white*, P1, P2), N-terminal inserts (dark blue, N1, N2) and aggregation-prone hexapeptide motifs (PHF6*, PHF6) in the repeat domain (orange, 1–4). hTau40^AT^ -expression is controlled by the murine Thy1.2 promoter. **b** Representative expression of hTau40^AT^ (Mr ~67 kDa) and endogenous mouse Tau (Mr ~ 45-55 kDa) in cortex, hippocampus, spinal cord and cerebellum of 2 months old hTau40^AT^ mice and age matched controls (WT) detected by the pan-Tau antibody K9JA. Note the slightly up-shifted human Tau in the spinal cord sample caused by hyperphosphorylation (red circle). ß-actin serves as loading control. **c** Quantification of (**b**). Ratio of hTau40^AT^ to endogenous mTau indicates the hTau40^AT^ expression level in different brain regions, e.g. cortex (~2x), hippocampus (~1x), spinal cord (~3x) and cerebellum (~1.5x). Each bar shows mean ± SEM of *n* = 17 animals. **d** Uniform distribution of hTau40^AT^ visualized by the human Tau specific antibody TauY9 in brain (cortex, hippocampus and cerebellum) and spinal cord of 2 months old hTau40^AT^ mice (d5-d8). Boxed areas with higher magnification (d9-d13) indicate mislocalization of hTau40^AT^ into cell bodies (*arrows*) and dendrites (*arrowhead*) and a strong immunoreactivity of mossy fibers in the CA3 hippocampal area (*asterisk*) in comparison to WT mice (d1-d4). WT: wild-type; A152T: hTau40^AT^ transgenic mouse strain; Ctx: cortex; Hippo: hippocampus; SpC: spinal cord; Cereb: cerebellum; SSCtx: somatosensory cortex; CA: cornu ammonis; F. nucleus: Fastigial nucleus; mo: months; Scale bars: 100 μm (d9, d10, d12, d13), 150 μm (d11), 300 μm (d1-d8)

To mimic pathological hallmarks of genetically provoked tauopathies, we created a novel transgenic mouse model expressing human full-length Tau (441aa, 2N4R) with the mutation A152T (hTau40^AT^), which has been linked to FTD spectrum disorders such as PSP [20] (Fig. 1a). Transgene insertion into the ROSA26-locus [14] under control of the neuron-specific murine Thy1.2 promoter [18] led to hTau40^AT^ expression at near-physiological levels in neurons of the brain and spinal cord. Heterozygous hTau40^AT^ protein levels were ~2-fold in cortex, ~1-fold in hippocampus, ~3-fold in spinal cord and ~1.5-fold in cerebellum in comparison to endogenous mouse Tau (mTau) (Fig. 1b, c). The uniform neuronal distribution of hTau40^AT^ was confirmed by stainings of brain and spinal cord sections using the human Tau specific antibody TauY9 (Fig. 1d). From 2 months of age onwards the hTau40^AT^ mice show an intense staining of CA3-mossy fibers (Fig. 1d11) as well as mislocalization of hTau40^AT^ protein into the somatodendritic compartment of cortical, hippocampal, spinal and cerebellar neurons (Fig. 1d9-10, 1d12-13).

### Co-aggregation of exogenous hTau40^AT^ and endogenous mouse Tau

**Fig. 2:**
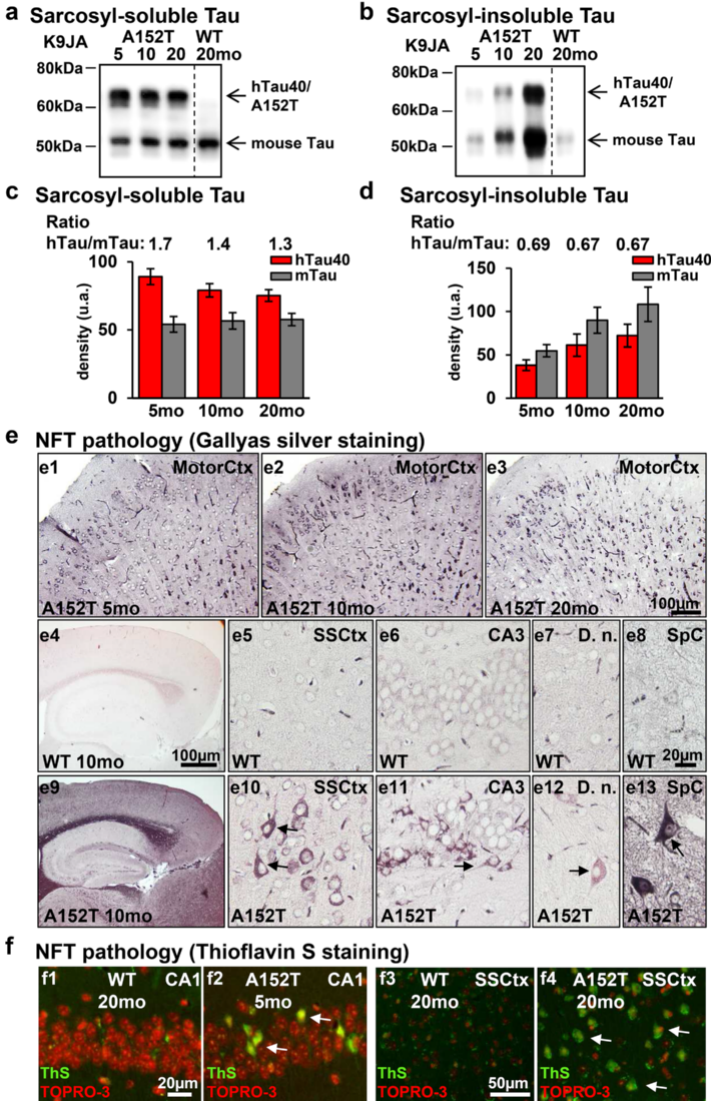
Progressive co-aggregation of endogenous mouse Tau and exogenous human hTau40^AT^ in mice. **a**-**b** Western blot analysis using the pan-Tau antibody K9JA shows sarcosyl-soluble and sarcosyl-insoluble Tau species of hTau40^AT^ (upper band; Mr ~67 kDa) and mouse Tau (lower band; Mr ~45-55 kDa). Note (**a**) the slight reduction of soluble hTau40^AT^ and (**b**) a progressive co-aggregation of insoluble mouse and human Tau in aging hTau40^AT^ mice. **c** Quantification of (**a**). Densitometric measurements of hTau40^AT^ (*red bars*) and mouse Tau (*grey bars*) indicate a reduction of soluble hTau40^AT^ with age (5-20mo) (note the declining ratio in aging transgenic mice). Each bar shows mean ± SEM of *n* = 4 animals. **d** Quantification of (**b**). Densitometric measurements of hTau40^AT^ (*red bars*) and mouse Tau (*grey bars*) indicate an increase of insoluble human hTau40^AT^ and mouse Tau with increasing age (5-20mo). Each bar shows mean ± SEM of *n* = 4 animals, error bars represent SEM. **e** Gallyas silver staining of different brain areas. (e1-e3) Progressive Tau aggregation in motor cortex of hTau40^AT^ mice with increasing age. Note NFTs with flame-shaped structure (*arrows*) in cortical (e10), hippocampal (e11), cerebellar (e12) and spinal (e13) neurons of 10 months old hTau40^AT^ mice, compared to silver-negative control (e4-e8). **f** Thioflavin S staining (*green*) and counterstaining with TOPRO-3 (*red*) of hippocampal CA1 pyramidal neurons (f2, *arrows*) and somatosensory cortical neurons (f4, *arrows*) confirms the presence of NFTs in hTau40^AT^ mice at 5 and 20 months of age and the absence of Tau-aggregates in WT mice (f1, f3). WT: wildtype; A152T: hTau40^AT^ transgenic mouse strain; SSCtx: somatosensory cortex; SpC: spinal cord; D.n.: Dentate nucleus; CA: cornu ammonis; mo: months; Scale bars: 20 μm (e5-e8, e10-e13, f1-f2); 50 μm (f3-f4); 100 μm (e1-e4, e9)

Pathological stages of Tau-aggregation can be classified into early conformational changes (antibodies Alz-50 or MC-1 [53]), early stages of aggregation (sarcosyl-insoluble Tau, “pretangles”), and fully developed neurofibrillary tangles (NFTs, visualized by Gallyas silver and Thioflavin S). In heterozygous hTau40^AT^ mice an age-dependent reduction of sarcosyl-soluble hTau40^AT^ was observed from 5 to 20 months of age (Fig. 2a, c). At 5 months, the ratio of soluble Tau species (transgenic hTau40^AT^ vs. endogenous mTau) was 1.7 and decreased to ~1.4–1.3 at 10–20 months of age (Fig. 2c). Sarcosyl-insoluble fractions of 5, 10 and 20 months old transgenic mice contained both, hTau40^AT^ and mTau, indicating a progressive co-aggregation of exogenous human and endogenous mouse Tau (Fig. 2b, d).

Gallyas-silver positive Tau inclusions detected in cortical, hippocampal, cerebellar and spinal neurons of hTau40^AT^ mice were considered as fully developed NFTs, exhibiting the typical flame-shaped structure (Fig. 2e9-13). In comparison non-transgenic littermates were silver-negative (Fig. 2e4-8). The formation of NFTs started as early as 3 months of age in cortical brain regions of hTau40^AT^ mice (data not shown) and the number of neurons containing NFTs increased in an age-dependent manner (Fig. 2e1-3). The presence of NFTs in hippocampal and cortical neurons of young and old hTau40^AT^ mice was confirmed by Thioflavin S stainings (Fig. 2f2, f4). One major neuropathological hallmark of PSP patients is the abnormal deposition of NFTs inside glial cells, the so called "tuft-shaped astrocytes" or "oligodendral coiled bodies" [23]. However, no Tau inclusions inside astrocytes or oligodendrocytes were detected in hTau40^AT^ mice from 5 to 20 months of age; similar to the lack of tufted astrocytes in a patient with A152T-mutated Tau [57].

### hTau40^AT^ induces pathological hyperphosphorylation and conformational changes of Tau at young age

**Fig. 3:**
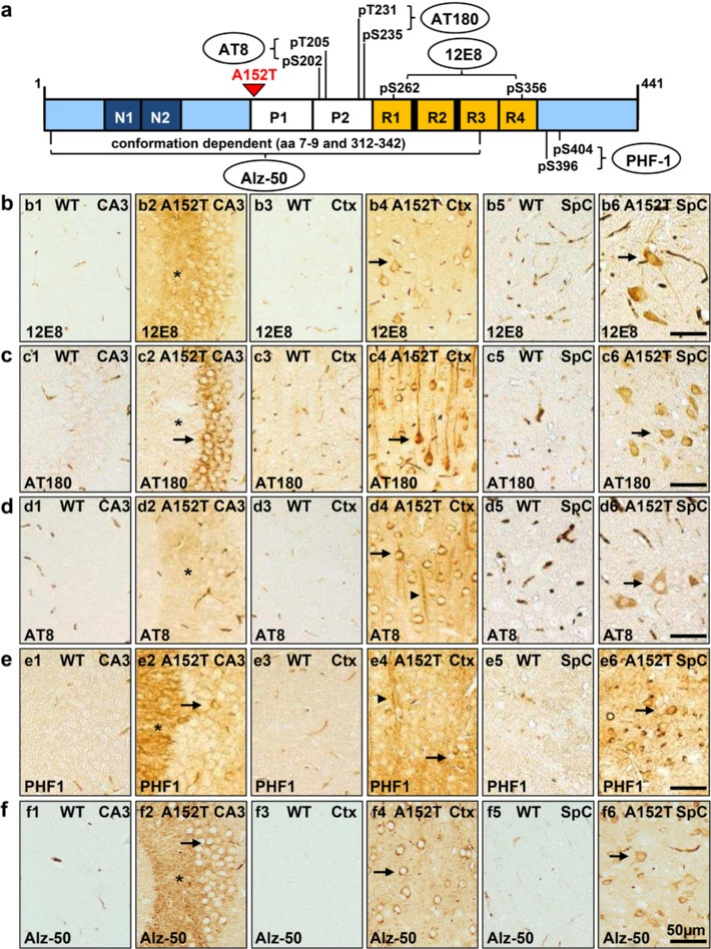
Expression of hTau40^AT^ induces pathological hyperphosphorylation and conformational changes of Tau in 3–5 months old hTau40^AT^ mice. **a** Illustration of human full length Tau with highlighted phospho-Tau antibody-epitopes AT180 (pThr231/pSer235), AT8 (pSer202/pThr205), 12E8 (pS262/pS356) and PHF1 (pSer396/pSer404) and pathological Tau conformation recognized by antibody Alz-50 (aa 7–9 + 312–342). **b**-**f** Hyperphosphorylated, mislocalized and conformationally changed Tau in brain and spinal cord of 3–5 months old hTau40^AT^ mice detected by antibodies: (**b**) 12E8, (**c**) AT180, (**d**) AT8, (**e**) PHF1 and (**f**) Alz-50. Note Tau missorting into the somatodendritic compartment of hippocampal, cortical and spinal neurons (*arrows*). Asterisks indicate localization of phosphorylated and conformationally changed Tau in the stratum lucidum. In WT mice, areas CA3, cortex and spinal cord are non-reactive for 12E8, AT180, AT8 and PHF1 (b-e1, b-e3, b-e5). Pathological Tau conformation identified by Alz-50 antibody in brain (f2 (asterisk = CA3-mossy fibers), f4) and spinal cord (f6) of hTau40^AT^ mice compared to WT littermates (f1, f3, f5). WT: wild type; A152T: hTau40^AT^ transgenic mouse strain; Ctx: cortex; SpC: spinal cord; CA: cornu ammonis; Scale bar: 50 μm (b1-f6)

**Fig. 4:**
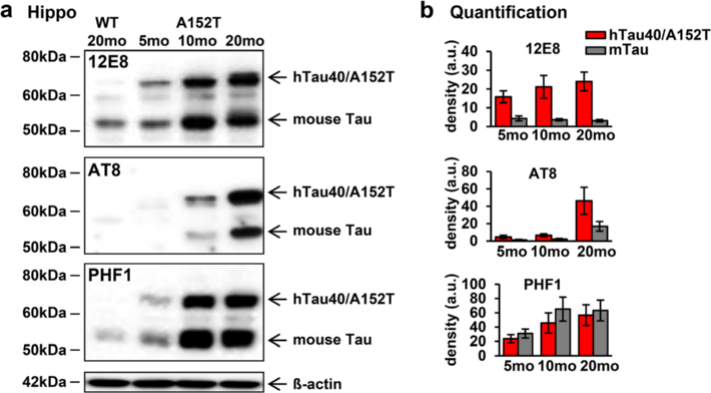
Progressive hyperphosphorylation of Tau in aging hTau40^AT^ mice (5-20mo). **a** Western blot analysis using phospho-Tau antibodies (12E8, AT8, PHF1) on hippocampal extracts of 5–20 months old hTau40^AT^ compared to 20 months old WT mice. **b** Quantification of (**a**). Densitometric analysis of hTau40^AT^ (*red bars*) and mouse Tau (*grey bars*) indicate an increase of hyperphosphorylated human hTau40^AT^ and mouse Tau in aging hTau40^AT^ mice (5-20mo). Each bar shows mean ± SEM of *n* = 4 animals. ß-actin serves as loading control. WT: wild type; A152T, hTau40^AT^ transgenic mouse strain; mo: months, a.u.: arbitrary units

In AD, PSP and other tauopathies, Tau undergoes hyperphosphorylation and conformational changes, accompanied by the translocation of pathological Tau from the axonal to the somatodendritic compartment of neurons [16, 70]. These changes are also observed in hTau40^AT^ mice as early as 3–4 months of age, *viz.* hyperphosphorylation [12E8 (pS262/pS356), AT180 (pT231/pS235), AT8 (pS202/pT205), PHF-1 (pS396/pS404)] and pathological conformation of Tau (Alz-50) appeared in hippocampal mossy fibers (asterisks, Fig. 3b2-f2), and in cell bodies and dendrites of hippocampal, cortical and spinal neurons (arrows, Fig. 3b2-f2, b4-f4, b6-f6), indicating missorting of pathological Tau in young hTau40^AT^ mice. The number of cortical neurons bearing hyperphosphorylated Tau (e.g. 12E8-, AT8- and PHF1-Tau) increased progressively with age (Additional file 1: Figure S1a-c). Analysis of hippocampal brain lysates confirmed the increase of hyperphosphorylated exogenous and endogenous Tau species with age (Fig. 4a-b). By contrast, the degree of phosphorylation at the 12E8 and PHF1 sites remained stable or decreased in 20 months old hTau40^AT^ mice, reflecting a potential correlation with progressive neuronal death (Fig. 9e-g).

### The A152T mutation correlates with pronounced neuroinflammation

**Fig. 5:**
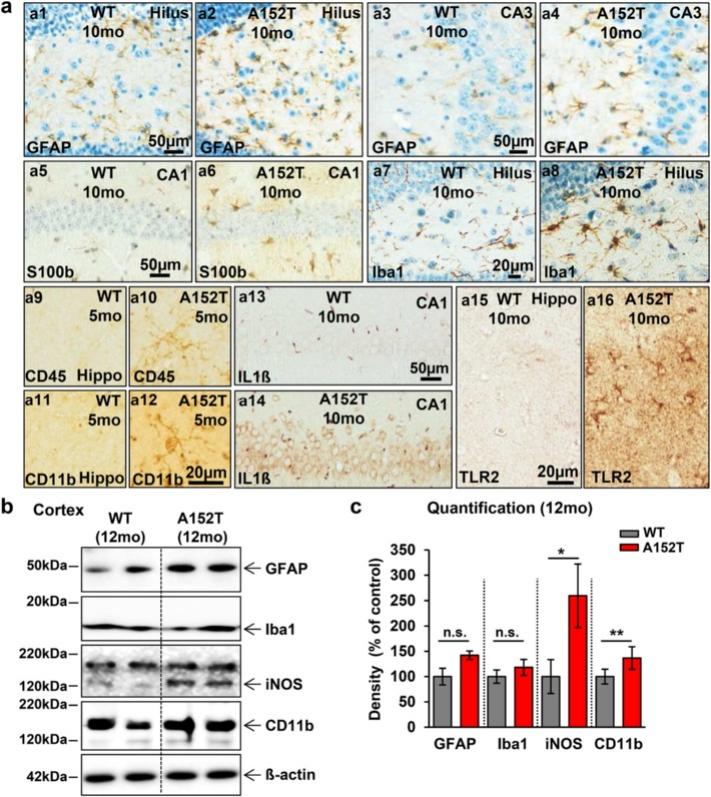
Activation of microglia and astrocytes in hTau40^AT^ mice. **a** Brain sections of hTau40^AT^ and WT mice stained with antibodies against GFAP (a1-4), S100b (a5-6), Iba1 (a7-8), CD45 (a9-10), CD11b (a11-12), IL1ß (a13-14) and TLR2 (a15-16) and partly counterstained with hematoxylin (*blue*). Prominent astrogliosis detected by GFAP and S100b occurs particularly inside the hippocampus formation, demonstrated by higher numbers of reactive astrocytes in hilus (a2), CA3 (a4) and CA1 (a6) of hTau40^AT^ mice, compared to aged-matched WT mice (a1, a3, a5). Iba1 staining indicates microgliosis in 10 months old hTau40^AT^ mice (a8) compared to WT (a7). Note that microglia of hTau40^AT^ mice show an activated non-phagocytic phenotype with retracted, thicker processes (a8) compared to resting microglial cells of WT mice (a7). Activated microglia are detected by CD45 (a10) and CD11b (a12) at 5 months of age in hTau40^AT^ mice, whereas WT mice show no reaction (a9 and a11). Additionally, 10 months old hTau40^AT^ mice show IL1ß-positive pyramidal CA1-neurons (a14) and TLR2-immunoreactive glial cells (a16) in the hippocampus, compared to non-reactive WT mice (a13, a15). **b** Western blots of cortical extracts indicate increased levels of GFAP, Iba1, iNOS and CD11b in hTau40^AT^ mice compared to WT mice. ß-actin serves as loading control. **c** Densitometric analysis of western blots (**b**) for inflammatory proteins (GFAP, Iba1, iNOS and CD11b), all normalized to ß-actin. The red bars indicate an increase of neuroinflammatory processes due to hTau40^AT^ -expression. Statistics: two-sided t test or Mann–Whitney-U-test indicate significant differences between WT and hTau40^AT^ mice (*:*p* < 0.05; **:*p* < 0.01). Each bar represents mean ± SEM of *n* = 4 animals. WT: wildtype; A152T: hTau40^AT^ transgenic mouse strain; CA: cornu ammonis; mo: months; IL1ß, interleukin 1 beta, TLR2, Toll like receptor 2; iNOS, inducible nitric oxide synthase; n.s., not significant; Scale bars: 20 μm (a7-12, a15-16), 50 μm (a1-6, a13-14)

Inflammatory processes play a major role in neurodegenerative diseases, but it is unclear whether neuroinflammation is the cause or the response to pathological events. Due to various toxic brain insults (e.g. aberrant protein deposition and neurodegeneration), microglial cells and astrocytes become activated, undergo morphological changes and change their protein expression pattern [72]. To address the question whether neuroinflammatory processes contribute to the pathogenesis of hTau40^AT^ mice, we probed brain sections with antibodies against several inflammatory proteins (Fig. 5). Relative to age-matched WT mice, 10 months old hTau40^AT^ mice show a prominent hippocampal and cortical astrocytosis and microgliosis with higher numbers of GFAP- and S100b-positive activated astrocytes and Iba1-positive microglia (Fig. 5a1-8). Although the microglia observed in the hippocampus (especially in the DG and CA3 regions) displayed an activated morphology with retracted, thicker processes (Fig. 5a8), only a few phagocytic microglial cells were detected in aged hTau40^AT^ mice (Additional file 1: Figure S6). The presence of activated microglia was confirmed by various microglial antigens, including CD45 and CD11b, from 5 months of age onwards (Fig. 5a10, a12). At 10 months, most CA1 neurons were positive for the pro-inflammatory cytokine IL1ß (Fig. 5a14). In addition, glial cells showed immunoreactivity against Toll-like receptor-2 (TLR2) (Fig. 5a16), indicating a potential activation of the TLR2-pathway.

By contrast, the majority of microglia in age-matched WT mice exhibited a ramified morphology with small somata and fine cellular processes (Fig. 5a7), characteristic of resting microglia. In addition, WT mice were immune-negative for microglia antigens (CD45, CD11b), pro-inflammatory cytokines (IL1ß) and TLR2 (Fig. 5a9,11,13,15). At 12 months of age, hTau40^AT^ mice demonstrated an overall increase of neuroinflammatory protein levels relative to age-matched WT mice in cortical brain extracts (Fig. 5c, GFAP: +40 %, Iba1: +20 %, iNOS: +150 %, CD11b: +40 %).

**Fig. 6:**
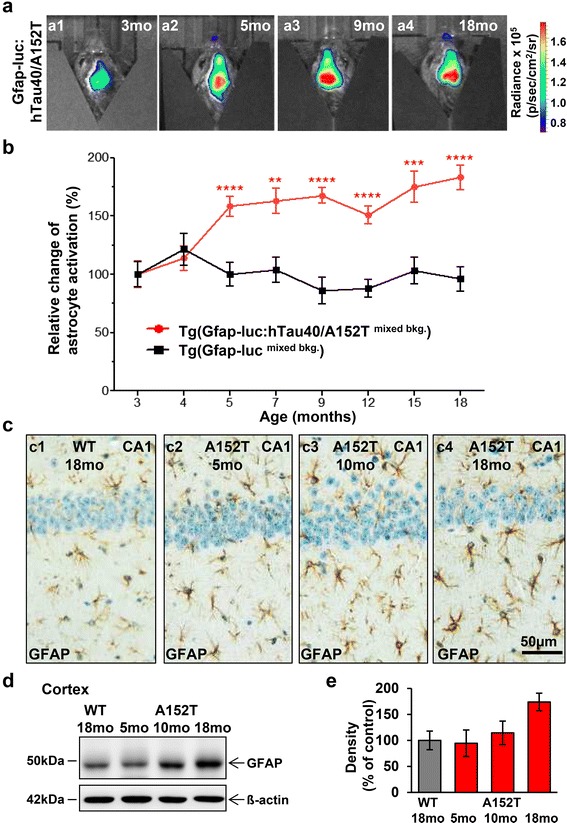
In vivo bioluminescence imaging (BLI) shows activation of astrocytes in response to aggregation of Tau. **a** Representative BLI signals emitted from the brains of Tg(Gfap-luc: hTau40^AT/mixed bkg^) mice in response to expression and aggregation of hTau40^AT^ with increasing age (3–18 months). **b** Change of astrocyte activation (%) as measured by in vivo BLI of luciferase activity. Data represents mean brain bioluminescence intensities (±SEM) obtained from Tg(Gfap-luc: hTau40^AT/mixed bkg^) mice (red circles, *n* = 18) in comparison to Tg(Gfap-luc^mixed bkg^) control animals (black squares, *n* = 9) at 3–18 months of age. Gfap-driven luciferase activity is strongly increased in Tg(Gfap-luc: hTau40^AT/mixed bkg^) mice from 5 months of age onwards in comparison to control groups, demonstrating an upregulation of astrocyte activation, which correlates with the aggregation of toxic hTau40^AT^. Two-way repeated measures ANOVA reveals highly significant group differences between Tg(Gfap-luc: hTau40^AT/mixed bkg^) and control groups (*p* < 0.0001, F_(2,199)_ = 33.46) and significant effect of interaction (*p =* 0.0001, F_(12,199)_ = 3.43) and time (*p =* 0.033, F_(6,199)_ = 2.33). Asterisks indicate levels of significance between Tg(Gfap-luc: hTau40^AT/mixed bkg^) mice and Tg(Gfap-luc^mixed bkg^) control animals as determined by post-hoc analysis. *:*p* < 0.05, **:*p* < 0.01, ***:*p* < 0.001, ****:*p* < 0.0001; mo, months of age; bkg, genetic background. **c**–**e** Progressive astrogliosis in hTau40^AT^ mice with increasing age. **c** Paraffin sections of 5 to 18 months old hTau40^AT^ and 18 months old WT mice were stained with GFAP (*brown*) and hematoxylin (*blue*). The majority of astrocytes of 18 months old hTau40^AT^ mice shows an activated morphology (c4) compared to aged matched WT mice (c1) and younger hTau40^AT^ mice (c2-3). **d** Western blot of cortical extracts demonstrates a progressive GFAP upregulation in hTau40^AT^ mice (time course 5, 10 and 18 months) compared to 18 months old WT mice. β-actin serves as loading control. **e** Densitometric analysis of western blot (**d**), normalized to ß-actin. The red bars indicate a progressive astrogliosis in hTau40^AT^ mice with increasing age compared to old WT mice. Each bar shows mean ± SEM of *n* = 3 animals. WT: wildtype; A152T: hTau40^AT^ transgenic mouse strain; CA: cornu ammonis; mo: months; Scale bar: 50 μm (c1-c4)

Progressive neuroinflammation in hTau40^AT^ mice was age-dependent as judged by increasing numbers of reactive astrocytes in hippocampus (Fig. 6c) and increasing amounts of GFAP protein levels in cortex brain extracts (Fig. 6d and e, GFAP: +70 % for 18 months old hTau40^AT^ mice relative to age-matched WT mice).

The upregulation of Gfap expression is a widely accepted marker of astrogliosis [72] and reporter strains optimized for non-invasive in vivo bioluminescence imaging (BLI) of neuroinflammation are available [reviewed in [46]]. In Tg(Gfap-luc) mice [117] luciferase is expressed upon induction of the murine Gfap promoter. Consequently, crossbreeding of Tg(GFAP-luc) mice with hTau40^AT^ mice allows longitudinal monitoring and quantification of astrocyte activation by in vivo BLI during the disease progression, similar to the monitoring of prion infectivity in prion-inoculated GFAP-luc mice [102].

At 3 months of age, hTau40^AT^ transgenic mice exhibited no overt signs of neuroinflammation as judged by histology (data not shown). Thus bioluminescence emission at 3 months of age was considered as background luciferase activity indicating the basal expression level of the Gfap promoter, which was defined as 100 %. At 5 months of age, bigenic Tg(Gfap-luc: hTau40^AT/mixed bkg^) demonstrated a significant rise in luciferase activity to 150 % as compared to the baseline Gfap promoter activity. After this initial upregulation, a further increase of Gfap-driven luciferase activity was observed in Tg(Gfap-luc: hTau40^AT/mixed bkg^) mice reaching a plateau of ~180 % at the age of 14 months. In contrast, luciferase activity in the brains of Tg(Gfap-luc^mixed bkg^) control animals remained stable over time (~100–120 %; Fig. 6a-b). The results demonstrate a strong upregulation of astrocyte activation in response to expression and accumulation of toxic hTau40^AT^.

**Fig. 7:**
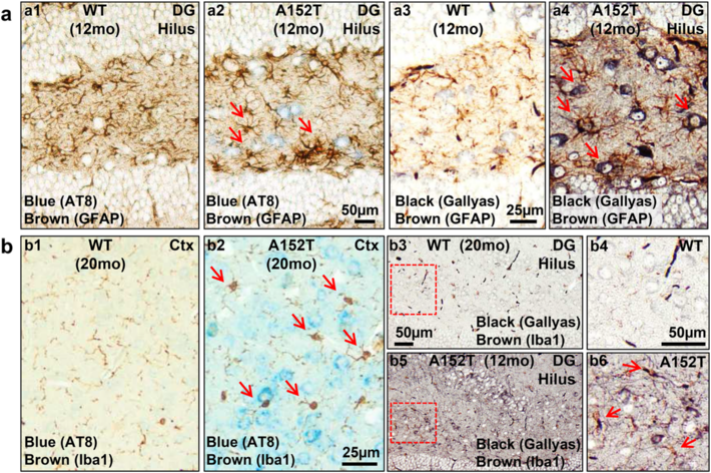
Inflammation vs. Tau pathology. **a**-**b** Paraffin sections of 12 months old hTau40^AT^ and WT mice were co-stained for neuroinflammation (GFAP or Iba1; *brown*) and Tau-phosphorylation (AT8; *blue*) or neuroinflammation (GFAP or Iba1, *brown*) and Tau-aggregation (Gallyas silver; *black*). Note the accumulation of glial cells in the surrounding of hippocampal neurons bearing pathological, AT8-phosphorylated Tau (a2 and b2, *arrows*) or NFTs (a4 and b5-6, *arrows*) in hTau40^AT^ mice. Age-matched controls show no AT8-staining, no NFTs and only a few astrocytes and microglia (a1, a3, b1, b3-4). Red-boxed areas (b3, b5) indicate close-ups (b4, b6). WT: wildtype; A152T: hTau40^AT^ transgenic mouse strain; CA: cornu ammonis; Ctx: cortex; DG: dentate gyrus; mo: months; Scale bar: 25 μm (a3-4, b1-2), 50 μm (a1-2, b3-6)

Co-stainings of neuroinflammation and Tau-phosphorylation or neuroinflammation and Tau-aggregation demonstrated the accumulation of astrocytes and microglia in the surrounding of AT8-positive and NFT-bearing neurons (Fig. 7a, b), indicating a direct correlation of Tau pathology and neuroinflammatory changes in hTau40^AT^ mice.

### Protein degradation systems are altered in hTau40^AT^ mice

Impaired protein quality control and impaired clearance via ubiquitin-proteasome and autophagosome-lysosome pathways are known to cause abnormal accumulation of disease-related proteins, which are deposited in intracellular or extracellular aggregates [21, 47, 71].

**Fig. 8:**
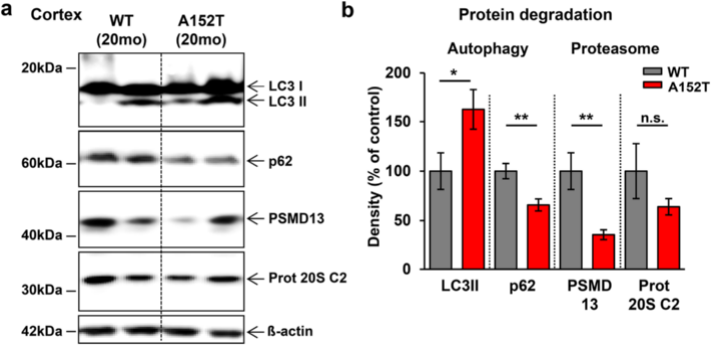
Expression of hTau40^AT^ alters protein degradation systems in aged mice. **a** Western blots of cortical extracts showing expression levels of autophagy- (LC3II, p62) and proteasome-related (PSMD13, proteasome 20S C2) proteins in 20 months old hTau40^AT^ and WT mice. β-actin serves as loading control. **b** Quantification of (**a**). Increased protein levels of LC3II (+60 %) and decreased protein levels of p62 (−35 %) clearly indicate an activation of autophagy and a reduction of proteasomal degradation (PSMD13, proteasome 20S C2: −40-60 %) in old hTau40^AT^ mice (*red bars*) compared to old WT mice (*grey bars*). Protein levels were normalized to β-actin. Each bar represents mean ± SEM of *n* = 5 animals. Statistics: two-sided t test indicates significant differences between WT and hTau40^AT^ mice (*:*p* < 0.05, **:*p* < 0.01). WT: wildtype; A152T: hTau40^AT^ transgenic mouse strain; mo: months; n.s., not significant

At 20 months of age hTau40^AT^ mice showed an activation of the autophagosome-lysosome pathway (Fig. 8b, LC3II: +60 %, p62: −35 %) and an inhibition of the ubiquitin-proteasome pathway (Fig. 8b, PSMD13 [regulatory subunit of the 26S proteasome]: −65 %, Proteasome 20S C2: −35 %) in cortex extracts as compared to age-matched WT littermates.

### Synaptic and neuronal loss in hippocampus and cortex of aged hTau40^AT^ mice

**Fig. 9:**
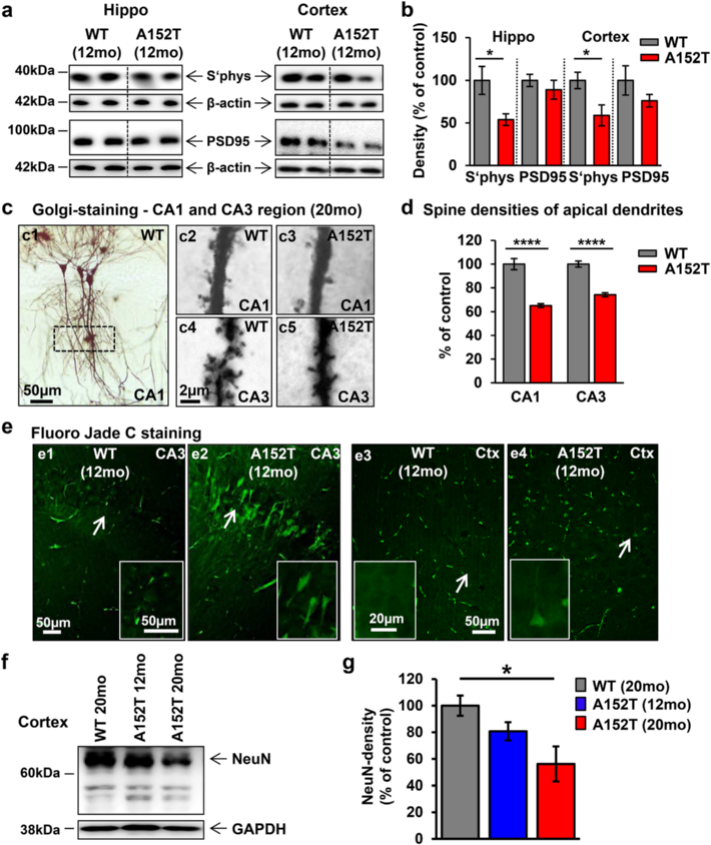
Synapse loss and neurodegeneration in hTau40^AT^ mice. **a** Western blots of hippocampus and cortex extracts showing expression levels of synaptic proteins (synaptophysin and PSD-95) of 12 months old hTau40^AT^ and WT mice. β-actin serves as loading control. Decreased levels of pre-synaptic (synaptophysin) and post-synaptic (PSD-95) markers are detected in hTau40^AT^ mice compared to WT. **b** Quantification of (**a**), normalized to ß-actin. Red bars indicate a reduction of pre-synaptic (synaptophysin) and postsynaptic (PSD-95) markers in hTau40^AT^ mice. Bars show mean ± SEM; *n* = 5. Statistics: two-sided t test indicates significant differences between WT and hTau40^AT^ mice (*:*p* < 0.05). **c** Dendritic spine density analyzed by Golgi-staining. (c1) Overview of Golgi-stained CA1 neurons of WT mouse with highlighted region of interest (*box*). (c2-c5) Higher magnification of CA1 and CA3 apical dendrites of hTau40^AT^ mice compared to WT at 20 months of age. Scale bar c1, 50 μm; c2-5, 2 μm. **d** Quantification of (**c**). A highly significant reduction of dendritic spines is detected in 20 months old hTau40^AT^ mice (red bar, CA1: ~35 %; CA3: ~25 %) compared to WT mice (*grey bars*). Bars show mean values ± SEM; *n* = 2-3 mice per group. Statistics: two-sided t test, ****:*p* < 0.0001. **e** Fluoro Jade C staining of floating sections from 12 months old hTau40^AT^ and WT mice to visualize neurodegenerative processes. Note the intense neurodegeneration in the CA3 region (e2) and cortex of hTau40^AT^ mice (e4) compared to WT mice (e1, e3). Arrows in the overviews indicate the location of higher magnified neurons (inserts). **f** Western blots of cortex extracts show NeuN expression levels in 12 and 20 months old hTau40^AT^ and WT mice. GAPDH serves as loading control. Note the dramatic loss of NeuN (especially ~66 kDa) in 20 months old hTau40^AT^ mice compared to 12 months old hTau40^AT^ and 20 months old WT mice. **g** Quantification of (**f**), normalized to GAPDH. Red bar indicates neuronal loss of ~40 % in 20 months old hTau40^AT^ mice compared to age-matched WT mice. Data represent mean values ± SEM; *n* = 4. Statistical comparisons were accomplished by one-way ANOVA followed by a post hoc Newman-Keuls multiple comparison test using Prism 5.0 (GraphPad Software). Asterisk indicates significant difference between WT and 20 months old hTau40^AT^ mice (*:*p* < 0.05). WT: wildtype; A152T: hTau40^AT^ transgenic mouse strain; CA: cornu ammonis; Ctx: cortex; mo: months; Scale bars: 2 μm (c2-5), 20 μm (inserts of e3-4), 50 μm (e1-4; inserts of e1-2)

One of the earliest changes occurring in AD is synaptic loss which correlates with the progression of cognitive decline in patients [106]. To check whether pre- and post-synaptic proteins were influenced by the accumulation of Tau in hTau40^AT^ mice, we probed hippocampal and cortical brain extracts of ~12 months old mice with antibodies against synaptic markers (Fig. 9a). This revealed a dramatic decrease of presynaptic synaptophysin (Fig. 9b, hippocampus: −50 %, cortex: −40 %), whereas post-synaptic PSD95 was less affected (Fig. 9b, hippocampus: −10 %, cortex: −35 %). The decrease of synaptophysin suggests a strong pathological effect of hTau40^AT^ at the pre-synapse rather than at the post-synapse. The mild defect of the post-synapse was consistent with spine counts of CA1-apical dendrites, which showed no significant differences in spine numbers between WT and cognitive unimpaired hTau40^AT^ mice at 10 months of age (Additional file 1: Figure S2a-b). By contrast, at more advanced age (20 mo) spine counts of cognitively impaired hTau40^AT^ mice demonstrated a highly significant decrease (reduction in CA1: −35 % and CA3: −25 %) compared to age-matched WT mice (Fig. 9c-d). Additionally, neurodegeneration and neuronal loss were observed in the hippocampus and cortex of hTau40^AT^ mice from 12 months onwards as visualized by Fluoro Jade C staining (Fig. 9e) and a reduction of NeuN protein levels at older ages (Fig. 9f-g; hTau40^AT^ mice: −40 %).

In conclusion, hTau40^AT^ mice show a strong defect of pre-synapses already at 12 months, whereas the post-synapse is only weakly affected at this stage, and damage becomes pronounced only at a later time point.

### Accumulation of hTau40^AT^ correlates with cognitive but not motoric deficits at advanced age

Transgenic hTau40^AT^ mice did not show abnormal motoric deficits up to 20 months of age. To show this, a detailed analysis was carried out at 10 and 16 months of age to characterize potential behavioral alterations caused by the expression and accumulation of hTau40^AT^. In the spinal cord, despite a ~3-fold overexpression of hTau40^AT^ and overt Tau-related pathological changes, we did not detect any motor deficiencies in a Rotarod test (Additional file 1: Figure S4) up to 16 months of age. In addition hTau40^AT^ mice did not exhibit any gait abnormalities in comparison to controls as determined by a Catwalk digital foot print analysis for single paw and inter-limb coordination (Additional file 1: Figure S5).

**Fig. 10:**
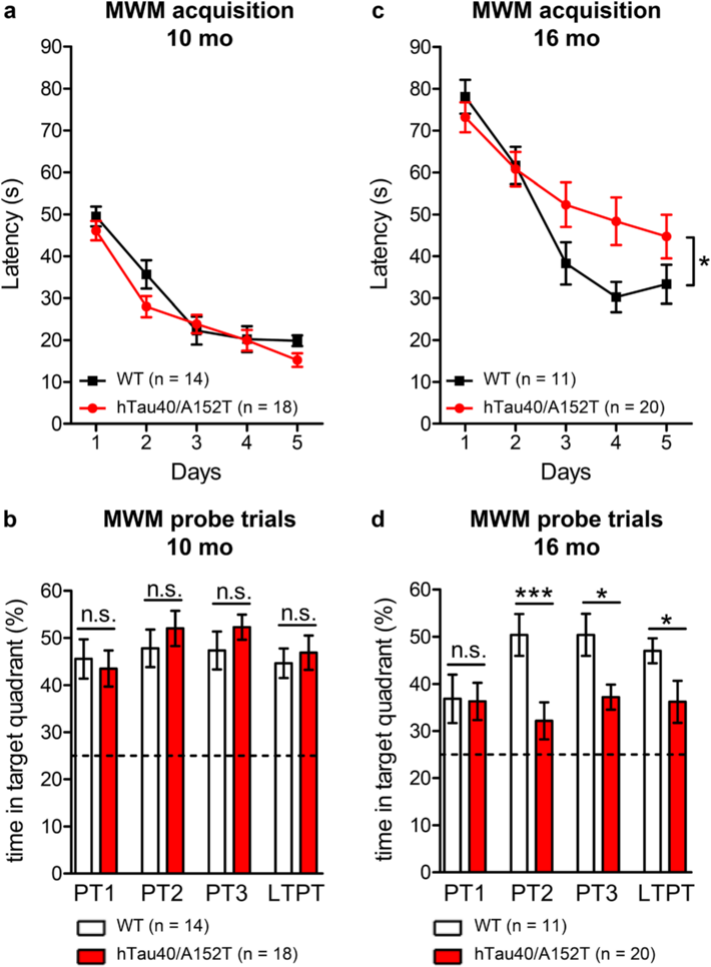
Cognitive impairment of hTau40^AT^ mice at 16 months of age. Morris water maze (MWM) test of 10 and 16 months old hTau40^AT^ mice in comparison to age-matched WT littermates. **a**, **b** At 10 months of age, spatial learning of hTau40^AT^ mice during a MWM acquisition is similar to WT controls and (**b**) hTau40^AT^ mice show a high preference for the target quadrant (>40 %), which is comparable to controls throughout all probe trials, indicating preservation of cognitive functions at 10 months of age. **c** In contrast, 16 months old hTau40^AT^ mice are impaired in learning the position of the hidden platform as demonstrated by significantly increased escape latencies during MWM acquisition in comparison to control mice (interaction effect, genotype x day, *p =* 0.033, F_(4,116)_ = 2.72). **d** Moreover hTau40^AT^ mice exhibit a lower preference for the target quadrant than WT animals from probe trial 2 onwards, pointing towards severe short- and long-term memory deficits due to accumulation of hTau40^AT^ at advanced age. **a**, **c** Data show mean escape latency (s) ± SEM for 10 months old WT (*n* = 14) vs hTau40^AT^ mice (*n* = 18) and 16 months old WT (*n* = 11) vs hTau40^AT^ mice (*n* = 20). Statistics: two-way repeated measure analysis of variances with post hoc Fishers LSD multiple comparisons test. Asterisks indicate a significant effect of interaction (genotype x day) between hTau40^AT^ mice and control group, *:*p* < 0.05. **b**, **d** Bars represent mean time in target quadrant (%) ± SEM for 10 months old WT (*n* = 14) vs hTau40^AT^ mice (*n* = 18) and 16 months old WT (*n* = 11) vs hTau40^AT^ mice (*n* = 20). Statistics: two-tailed Students t-test between hTau40^AT^ mice and control group for each probe trial, *:*p* < 0.05, ***:*p* < 0.001; n: number of mice; WT: wild-type animals; mo: months of age

The cognitive performance of hTau40^AT^ mice was investigated using a Morris water maze (MWM) test. Swimming speed did not differ from WT controls at 10 and 16 months of age, confirming normal motor abilities of the animals (Additional file 1: Figure S3). Regarding spatial learning and memory, hTau40^AT^ mice were indistinguishable from age-matched controls at 10 months of age, demonstrating intact cognitive function at early Tau pathological stages (Fig. 10a, b). However, at 16 months there was pronounced impairment of spatial learning. In comparison to controls, hTau40^AT^ mice were considerably slower to reach the hidden platform during MWM acquisition as demonstrated by an increased escape latency and path length (interaction effect genotype x day for escape latency: *p* = 0.033, F_(4,116)_ = 2.72; interaction effect genotype x day for path length: *p* = 0.027, F_(4,116)_ = 2.88) (Fig. 10c). In addition, probe trials conducted at days 3, 4, 5 and 2 days after finishing the acquisition phase (long-term probe trial) revealed severe short- and long-term memory impairments of 16 months old hTau40^AT^ mice in comparison to WT mice, as indicated by a reduced preference for the target quadrant (Fig. 10d, Additional file 1: Figure S3f).

**Fig. 11:**
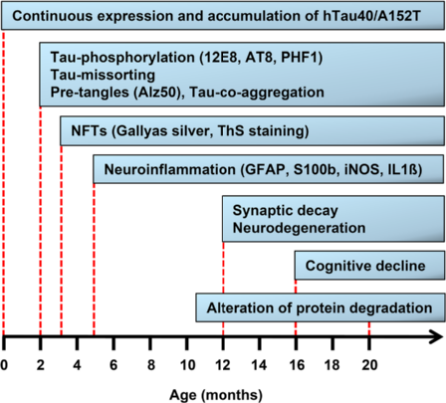
Diagram of progression of neuropathology in hTau40^AT^ mice. Continuous expression and accumulation of hTau40^AT^ in brain and spinal cord of hTau40^AT^ mice lead to early pathologic changes at 2–3 months of age, including Tau-phosphorylation, Tau-mislocalization into the somatodendritic compartment and Tau-co-aggregation of endogenous mouse Tau and exogenous hTau40^AT^ into pretangles. At 3–4 months of age, initial mature tangles (NFTs) are detected by Gallyas silver and ThS staining. Neuroinflammatory processes start from 5 months onwards. Progressive Tau-phosphorylation, Tau-co-aggregation and neuroinflammation likely cause neurotoxic events, resulting in synaptic decay, neurodegeneration and cognitive decline of aged hTau40^AT^ mice. In parallel, progressive neuroinflammation might alter protein degradation systems, inducing autophagy and reducing proteasome activity in aging hTau40^AT^ mice. mo: months

Taken together, the accumulation of endogenous and exogenous mutant Tau in hTau40^AT^ mice in the hippocampus correlates with impairment of spatial learning and memory at ~16 months of age, which parallels the overall increase of brain inflammation, synapse loss and neuronal death. In addition the loss of cognitive function correlates with the loss of synapses and pyramidal neurons in the hippocampus which was initially observed with ~12 months of age (Fig. 11).

## Discussion

In recent years several Tau mutations have been identified as genetic risk factors for FTDP-17 [36, 48]. Most of the mutations are located in the repeat domain (microtubule binding region; e.g. P301L, P301S and ΔK280) and reduce the Tau-MT-interaction and MT-assembly and/or increase Tau-aggregation by enhancing the ß-propensity of Tau. This results in Tau-hyperphosphorylation, missorting and aggregation [42, 109]. By contrast, mutations in the N-terminal Tau projection domain (i.e. R5L, G55R), which are partly related to PSP [R5L, [86]], and their underlying mechanisms are not well understood at present. However, N-terminal mutations may impact on the 3D-structure of Tau (e.g. the "paperclip conformation", [52]) or influence intra- and intermolecular interactions that might affect MT and neuronal cell functions [51]. The *MAPT* p.A152T variant was recently recognized as a risk factor for several FTD spectrum disorders including AD, highlighting the clinical and pathological variability associated with *MAPT* p.A152T [20, 38, 55, 57, 60]. To address the question of how p.A152T Tau induces neuropathological features including cognitive decline, we generated a novel transgenic mouse model expressing human full-length Tau with mutation A152T (hTau40^AT^).

### Neuropathology of hTau40^AT^ mice in comparison to AD- and PSP-cases and Tau transgenic mouse models

In spite of the low, near-physiological transgene expression, hTau40^AT^ causes Tau-hyperphosphorylation at several diagnostic sites (e.g. AT8, PHF1) and Tau-missorting of transgenic and endogenous Tau into the somatodendritic compartment of neurons starting at early age (Figs. 3, 4, Additional file 1: Figure S1). The results are comparable to tauopathy mouse models with FTDP-17-related Tau-mutations [30, 62, 75, 87, 92] and to pathological reports of AD- and PSP-cases [16, 76].

Furthermore *MAPT* p.A152T increases the hydrophilic character of Tau and generates an additional phosphorylation site just upstream of the TP-motif 153–154 [57], which is one of numerous targets in Tau by proline-directed kinases (i.e. MAPK, cdk5 and GSK3). This type of phosphorylation is characteristically increased in most tauopathies, and indeed, the phosphorylation of T153 is observed both in cell models and AD brain [5, 49]. Thus the mutation Ala→Thr in the preceding residue 152 could impact the phosphorylation pattern and function of this region of Tau.

In vitro, hTau40^AT^ shows a somewhat reduced tendency to form Tau-fibers and an increase in oligomers [20]. However, in mice the exogenous hTau40^AT^ is able to interact with endogenous mouse Tau to form stable co-aggregates and flame-shape NFTs (Fig. 2), comparable to transgenic mice expressing human Tau variants with mutations in the repeat domain or C-terminus, such as G272V, ΔK280, P301S, P301L and R406W [3, 75, 91, 104, 116].

Neuropathologically, Tau-co-aggregates detected in hTau40^AT^ mice resemble neuronal NFTs of AD-patients rather than the spherical tangles of PSP-patients [13, 17, 105]. Furthermore "tufted astrocytes" containing abnormal Tau deposits, a classical marker of PSP [12], are lacking in mice and in patients with mutated hTau40^AT^ [57].

Although hTau40^AT^ mice develop remarkable Tau phosphorylation and aggregation in spinal and motor cortical neurons (Figs. 2 and 3), motor functions were preserved (Additional file 1: Figures S4, S5). This is in contrast to earlier generations of Tau-transgenic mice with high overexpression of mutant Tau in the CNS including spinal cord, which show strong motor deficits and thus interfere with cognitive assays such as MWM and others [61, 62, 96, 107, 116]. However, the hTau40^AT^-expression (due to a single copy in the ROSA26-locus) seems to be low enough to avoid motor deficits in hTau40^AT^ mice and thus to allow cognitive testing, similar to other mouse models with rather near-physiological levels of Tau transgene expression in the spinal cord [82, 92].

From our results we conclude that the expression of A152T-Tau (a Tau-variant mutated in the N-terminal half, near the proline-rich region) leads to hyperphosphorylation, missorting and aggregation of Tau, similar to pathological features of FTDP-mouse models expressing human Tau-variants with mutations in the C-terminal half that are known to affect Tau-microtubule interactions and Tau-aggregation [91, 92, 116]. How this A152T-Tau promotes tauopathy (e.g. by impacting the 3D-structure of Tau, alterations in MT dynamics, phosphorylation pattern, sensitivity to proteases etc.) will require further investigation. But the proximity to the central proline-rich domain which harbors interaction sites with proteins with SH3 domains [40] is suggestive of a role in altered signaling pathways. In this context it is interesting to note that the trans-cellular spreading of Tau-pathology appears to be mediated by high MW non-fibrillar oligomers of Tau with pronounced hyperphosphorylation by proline-directed kinases [101].

### Inflammatory response versus hTau40^AT^ -pathology

Neuroinflammation plays a crucial role in the development and progression of neurodegenerative diseases, but it is still a matter of debate whether inflammatory processes trigger the pathology (e.g. Tau-aggregation) or whether activated glial cells react to the aggregation of toxic proteins in their surrounding [2]. The hTau40^AT^ mice develop a remarkable neuroinflammatory response as judged by the activation of astrocytes and microglia and the upregulation of inflammation-related proteins, starting from 5 months onwards (Figs. 5 and 6), similar to reports of patients with chronic neurodegenerative diseases including AD, PSP, PD, ALS and prion disease [1, 31, 43, 83].

Microglia cells are locally activated by misfolded proteins (e.g. Aß and alpha-synuclein) [83], in particular by structural changes which activate toll-like receptors (TLR) and by expression of pro-inflammatory cytokines (e.g. IL-1ß) or inflammation-induced enzymes like iNOS [28, 45]. Apart from glial cells, neurons can serve as source of cytokines (i.e. IL-1ß), and express inducible nitric oxide synthase (iNOS) in pathological conditions [43, 44], as shown here for CA1-neurons of hTau40^AT^ mice (Fig. 5).

Similar to the accumulation of activated glial cells in the surrounding of NFT-bearing neurons in hTau40^AT^ mice (Fig. 7), activated microglia are also associated with Aß-plaques in AD patients and transgenic APP-23 mice [93, 97]. Furthermore, misfolded α-synuclein activates directly microglia with a classical cytokine upregulation, morphological changes and alterations in TLR gene expression [11]. Reports on transgenic mice (expressing aggregation-prone Tau variants) strengthen the linkage between the accumulation of intracellular aggregated Tau species and an inflammatory response [10, 75, 116]; whilst the expression of an anti-aggregant Tau variant “protects” mice against a pronounced gliosis [75].

Experiments with implanted IL-1-releasing pellets into rat brains, leading to Tau-hyperphosphorylation, have suggested a function of IL-1 as trigger for the progression of neurofibrillary pathology in AD [39, 94]. Furthermore, the proinflammatory cytokine TNF initiates the accumulation of Tau preferentially in neurites via reactive oxygen species [37]. Conversely, neuroinflammation and Tau pathology were diminished in PS19 mice by treatment with the anti-inflammatory compound FK506 [116]. Gene expression profiles of rTg4510 mice suggest a correlation of Tau-pathology, cognitive decline and upregulation of inflammatory genes similar to AD cases [111]. All of these observations point to a tight relationship between Tau toxicity and pro-inflammatory cytokines.

In patients with AD or PSP, ongoing gliosis is visualized and quantified by positron emission tomography (PET) and usually increases with the disease stage in affected regions [85, 118]. In comparison, in aging bigenic mice (Gfap-luc:hTau40^AT/m.bkg^) the rise of GFAP-dependent luciferase activity reflects the kinetics of astrocyte activation in vivo as monitored by bioluminescence imaging (Fig. 6), confirmed by staining for inflammation markers (Figs. 5 and 6). This underscores the potential of BLI to characterize progressive neurodegenerative diseases (e.g. AD and ALS) [46]. In hTau40^AT^ mice the neuroinflammation precedes cognitive decline (Figs. 5, 6, 7, and 10). Based on the probable link between neuroinflammation, Tau-pathology and cognitive failure, BLI of bigenic (Gfap-luc: hTau40^AT/mixed bkg^) mice may be used to validate and improve treatment strategies and define time points of early therapeutic intervention before onset of behavioral deficits.

From our results, we conclude that neuroinflammation in hTau40^AT^ mice occurs in response to early Tau-phosphorylation and Tau-aggregation at young age (Figs. 2 and 3). At later stages, neuroinflammatory changes might further boost Tau pathology and neurotoxic processes, which finally lead to synaptic damage and neuronal death (Figs. 9 and 10).

### Accumulation of hTau40^AT^, neuroinflammation and protein degradation: a reciprocal relationship

Aberrations in protein clearance systems contribute to the pathogenesis of neurodegeneration, causing the accumulation and aggregation of disease-relevant proteins [47, 59, 74, 80]. In the present study, 20 months old hTau40^AT^ mice show a reduced proteasome activity and an upregulation of the autophagosome-lysosome pathway (Fig. 8), pointing towards a cross-talk between both protein degradation pathways, as suggested by others [56]. In this regard, provoked proteasome impairments (by drugs or gene silencing) induce autophagy as a compensatory protein degradation pathway in various cell models [26, 65, 81]. Similar changes occur after lipopolysaccharide (LPS)-induced neuroinflammation, which decreases proteasome activity and causes hippocampal neurodegeneration [84]. Moreover, autophagy can be upregulated by immune signaling molecules (e.g. TLRs) or the IL1R pathway [95, 98]. Therefore, the observed changes in the protein degradation systems in the aged hTau40^AT^ mice are likely explained by the progressive neuroinflammation (Figs. 5, 6, 7 and 8).

Conversely, the activation of autophagy would appear as a promising strategy against neurodegeneration, since aggregated proteins (e.g. Tau) are preferentially degraded by chaperone-mediated autophagy [110]. Furthermore, application of autophagy inducers (e.g. rapamycin, trehalose) lowers Tau-hyperphosphorylation and neuroinflammation and improves cognition in transgenic mouse models [29, 68, 89]. Beyond that, trehalose suppresses Tau-aggregation in an inducible N2a cell model of Tau pathology and diminishes cytotoxicity [58]. However, the activation of autophagy in hTau40^AT^ mice occurs only after NFT formation and onset of neuronal loss, obviously too late to rescue Tau induced toxicity and cognitive phenotype (Figs. 2, 8, 9 and 10), similar to old 3xTg-AD mice with NFTs showing no cognitive recovery after autophagy-activation [69].

### Synaptic decay and neuronal loss parallel cognitive impairments in hTau40^AT^ mice

Synapse loss is a fundamental correlate of cognitive decline in AD [106]. Our data indicate an age-dependent failure of learning and memory capacities in hTau40^AT^ mice. At 10 months, when dendritic spine densities are still unaffected, hTau40^AT^ mice are still cognitively normal (Additional file 1: Figure S2; Fig. 10). However, 12 months old hTau40^AT^ mice show already incipient “synaptotoxicity” observed by reduced levels of synaptic proteins (especially presynaptic markers). Decreased level of synaptophysin (Fig. 9a-b), presumably caused by the accumulation of toxic hTau40^AT^ inside presynaptic termini, might serve as a first indicator for pathological changes in the synaptic connectivity, as described for other Tau transgenic mice [75, 108, 116]. From 12 months onwards progressive neurodegeneration (Fig. 9e) destroys cognitive pathways, resulting in severe learning and memory deficits of 16 months old hTau40^AT^ mice (Fig. 10c-d). Consistent with this, cognitively impaired ~20 months old hTau40^AT^ mice show a profound loss of CA1- and CA3-synaptic spines (Fig. 9c-d), similar to other tauopathy mouse models, where synapse loss was correlated to cognitive decline [87, 92, 100, 108].

### Toxic gain and loss of function in hTau40^AT^ mice affects cognition

The mechanisms leading to Tau-dependent neurotoxicity in AD- or FTDP-17-patients remain unclear in detail, but there is evidence that a combination of toxic gain of function and the loss of normal Tau function serves as a trigger for neurodegenerative processes [112]. Under physiological conditions non-mutated Tau stabilizes MTs in axons, but post-translational modifications (e.g. phosphorylation) could alter its interaction with MTs and other cell components and thus might result in impairment of axonal transport, Tau-missorting and formation of PHFs [70]. By contrast, hTau40^AT^ shows a reduced MT affinity and slower aggregation in vitro, but exhibits an enhanced tendency to form Tau oligomers [20]. Whether the *MAPT p.A152T* mutation alters the function of the Tau projection domain as MT-spacer [19] or its interaction with the dynactin complex [67], histone deacetylase 6 [24], tyrosine kinases such as Fyn [50] or other proteins is still unknown. Further intramolecular changes of hTau40^AT^ impacting the 3D-structure cannot be excluded. Since transgenic mice, expressing aggregation-prone Tau proteins, develop early cognitive decline [87, 92, 100], the co-aggregates of hTau40^AT^ mice (Fig. 2) might have less beta structure and might be less neurotoxic, postponing memory loss in hTau40^AT^ mice to older age (Fig. 10).

A proper function of the ubiquitin-proteasome system (UPS) is essential for correct synaptic transmission, since the UPS operates in the pre- and postsynaptic compartment by regulating neurotransmitter release, synaptic vesicle recycling and the dynamic behavior of the PSD and dendritic spines [114]. Proteasome inhibition causes impairment of neuronal protein synthesis [25], loss of synaptic proteins [7] and LTP impairments [27]. Furthermore bilateral hippocampal injection of lactacystin (proteasome inhibitor) produces retrograde amnesia in rats [64]. In this context, reduced proteasome activity in old hTau40^AT^ mice (Fig. 8) might impact on synaptic function and trigger synapse loss (Fig. 9) and cognitive decline (Fig. 10).

Neuroinflammatory processes interfere with learning and memory and are related to synaptic decay and neuronal loss in neurodegenerative disorders [66, 115]. Although physiological levels of IL-1ß are beneficial for synaptic plasticity [6], IL-1ß-treatments of cultured neurons induced a marked loss of synaptic connections [73]. In astrocytes, elevated levels of IL-1 trigger the overexpression of the neurotrophic cytokine S100b, a calcium binding protein. S100b increases the free calcium concentration in neurons; whereas mice overexpressing S100b show enhanced excitotoxicity, altered synaptic plasticity and cognitive impairment [9, 34, 78]. Since neuroinflammation is a major hallmark of hTau40^AT^ mice (Figs. 5, 6 and 7), the presence of activated glial cells and inflammatory proteins might serve as major trigger for cognitive decline.

## Conclusions

In summary, the present study shows that expression of hTau40^AT^ at low near-physiological levels is sufficient to induce a severe neuropathology leading to functional deficits and neuronal death in vivo (Fig. 11). Our results support the hypothesis that the rare *MAPT p.A152T* mutation promotes a neurotoxic gain of function, most likely triggered by the enhanced neuroinflammation and excitotoxic events. Thus the new hTau40^AT^ mouse model is suitable for further mechanistic studies of Tau induced neurotoxicity and for in vivo validation of compounds covering Tau-pathology and neuroinflammation.

## Additional file

Additional file 1:
**Supplemental material and results.** (PDF 5946 kb)

## Abbreviations

Aβ: Amyloid-beta
AD: Alzheimer disease
ALS: Amyotrophic lateral sclerosis
Bkg: background
BLI: bioluminescence imaging
bvFTD: behavioral variant of frontotemporal dementia
CA: cornu ammonis
CBD: corticobasal degeneration
CNS: central nervous system
ΔK280: Tau mutant with deletion of lysine 280
DG: dentate gyrus
ES: embryonic stem cells
FTD: frontotemporal dementia
FTDP-17: frontotemporal dementia and parkinsonism linked to chromosome 17
FTD-s: frontotemporal dementia spectrum disorders
GAPDH: Glyceraldehyde 3-phosphate dehydrogenase
GFAP: glial fibrillary acidic protein
HD: Huntington Disease
HSC70: heat shock cognate protein 70
hTau40: human full-length Tau (2N4R, 441 residues)
hTau40^AT^: hTau40 with mutation A152T
iNOS: inducible nitric oxide synthase
LC3: microtubule-associated protein 1 light chain 3
mf: mossy fibers
mo: months
MT: microtubule
MWM: Morris water maze
NeuN: neuronal nuclear antigen
NFT: neurofibrillary tangle
NMDAR: N-methyl D-aspartate receptor
PET: positron emission tomography, PHF, paired helical filament
Prot 20S C2: C2 subunit of the 20S proteasome
PrP: prion protein
PSD95: postsynaptic density protein 95
PSMD13: a regulatory subunit of the 26S proteasome
PSP: Progressive Supranuclear Palsy SSCtx, somatosensory cortex
Tau^ΔK^: full-length human Tau (2N4R, residues 1–441) with ΔK280 deletion mutation
TauRD^ΔK^: Tau 4-repeat domain (construct K18, residues 244–372) with ΔK280 mutation
hTau40^AT^: full-length human Tau (2N4R, residues 1–441) with A152T mutation
Tg: transgenic
UPS: ubiquitin-proteasome system
WT: wild type
3D: three dimensional
