# Supplementary material for: Age-dependent neuroinflammation and cognitive decline in a novel Ala152Thr-Tau transgenic mouse model of PSP and AD
Source: Acta Neuropathol Commun. 2016 Feb 25;4:17. doi: 10.1186/s40478-016-0281-z (PMC4766625; doi:10.1186/s40478-016-0281-z)

**Manuscript: ANEC-D-16-00011, Supplemental material and results**

# Age-dependent neuroinflammation and cognitive decline in a novel Ala152Thr-Tau transgenic mouse model of PSP and AD

**Authors:**
Astrid Sydow[1,2*†], Katja Hochgräfe[2†], Stefanie Könen[1], Daniela Cadinu[2], Dorthe Matenia[1], Olga Petrova[1], Maria Joseph[1,2], Frank Johannes Dennissen[1], Eva-Maria Mandelkow[1,2,3*]

**Addresses:**
(1) German Center for Neurodegenerative Diseases (DZNE), Ludwig-Erhard-Allee 2, 53175 Bonn, Germany
(2) Max-Planck-Institute for Metabolism Research, Hamburg Outstation, c/o DESY, Notkestrasse 85, 22607 Hamburg
(3) CAESAR Research Center, Ludwig-Erhard-Allee 2, 53175 Bonn, Germany

**† Equal contributors**

*** Correspondence:** sydow@mpasmb.desy.de; mandelkow@mpasmb.desy.de

## Supplement Material & Supplement Methods:

***Rotarod test:***
Mice were placed on a turning rod (3.2 cm in diameter; MedAssociates) with their body axis perpendicular to the rotation axis. The head was directed against the direction of the rotation such that the animal had to progress forward to maintain its balance. Five trials with an inter trial interval of 45min were carried out. Trials 1 and 2 were performed at slow, constant speed (16 rpm) for a maximum duration of 180s. Trials 3-5 were performed with the accelerating rod (4rpm-40rpm within 5min) for a maximum duration of 5min. On the following day, a $6^{th}$ trial with the accelerating rod was carried out. The performance of the mice was evaluated by scoring the latency to fall down. All data represent group mean values with standard error of the mean (SEM). Statistical comparisons between transgenic mice and controls were performed by an unpaired two-tailed Student´s t-test using Prism 5.0 (GraphPad Software). The accepted level of significance was $p<0.05$.

***CatWalk test:***
A detailed gait analysis was performed using the CatWalk XT 10.5 system (Noldus). Transgenic $hTau40^{AT}$ mice (n=14, mixed genders) were compared to age-matched control littermates (mixed genders, n=14) at the age of 11 months. Briefly, the CatWalk system consists of an enclosed walkway on a glass plate that is traversed by the mouse. Green light enters at the long edge of the plate and is completely internally reflected. In case a mouse's paw contacts the glass surface, light is scattered downwards resulting in a sharp and bright digital image of a paw print. The whole run is tracked by a video system placed under the glass plate. Mice were trained to cross the glass plate in three consecutive trials. The number of compliant runs was n=3 with a minimum duration of 0.5s and a maximum duration of 3s. The following parameters were analyzed: (a) intensity of the paw print (arbitrary units, a.u.), expressing the mean pressure of each paw during the floor contact; (b) stride length (cm), describing the distance between two consecutive paw placements of a particular paw; (c) swing (s),measuring the time between one step and the next for a particular paw; (d) stance (s), indicating the amount of time a particular paw rests on the walkway; (e) step cycle (s), exhibiting the time between two consecutive paw placements (stance + swing duration); (f) duty cycle (%) showing the ratio between stance duration and step cycle duration; (g) base of support (cm) representing the distance between the front or the hind paws; (h) regularity index (RI, %) as a measure of interlimb coordination defined by the percentage of normal step sequence pattern in relation to the total number of paw placements.

**Supplement figure legends:**

**Fig. S1: Progressive Tau-phosphorylation in aging hTau40$^{AT}$ mice (5-20mo).**
**(a-c)** Paraffin sections of 5-20 months old hTau40$^{AT}$ and WT mice were stained with (a) 12E8, (b) AT8 or (c) PHF1 and hematoxylin (blue). Hyperphosphorylated Tau progressively accumulates with increasing age in hTau40$^{AT}$ mice (12E8 (a2-a4), AT8 (b2-b4), PHF1 (c2-c4). Note mislocalization of hyperphosphorylated Tau in the somatodendritic compartment of cortical neurons (arrows). (a4, b4, c4) The intense staining of neuropil in older hTau40$^{AT}$ mice clearly indicates an increased phosphorylation of axonal extensions. (a1, b1, c1) By contrast old WT mice show no immunoreactivity for all tested Tau phospho-epitopes. WT: wildtype; A152T: hTau40$^{AT}$ transgenic mouse strain; SSCtx; Somatosensory cortex; MCtx, Motor cortex; mo: months; Scale bar: 50µm (a1-c4).

**Fig. S2: No spine loss in hTau40$^{AT}$ mice at 10 months of age.**
**(a)** Dendritic spine density analyzed by Golgi-staining. Representative images of apical dendrites of CA1-hippocampal neurons of 10 mo old WT and hTau40$^{AT}$ mice. Scale bar: 3µm.
**(b)** Quantification of (a). No significant differences of the CA1 apical dendritic spine density are detected between 10 months old hTau40$^{AT}$ mice (red bar) and age-matched WT mice (grey bars) by two-sided t test. Bars show mean ± SEM. n.s, not significant.

**Fig. S3: Morris water maze test shows learning and memory deficits of hTau40$^{AT}$ mice at 16 months of age.**
**(a)** While hTau40$^{AT}$ mice display unaltered learning behavior at 10 months of age, **(b)** 16 months old hTau40$^{AT}$ mice exhibit significant learning impairments in comparison to age-matched controls as indicated by increased path length during Morris water maze acquisition (interaction effect, genotype x day, p=0.027, $F_{(4,116)}$=2.88).
**(c-d)** Swimming speed does not differ between hTau40$^{AT}$ and control mice (p>0.05 for all comparisons) at 10 months and 16 months of age, excluding motor disabilities as underlying cause for different learning rates. (a-d) Data shows mean path length (cm) or swim speed (cm/s) ± SEM. Statistics: two-way repeated measure analysis of variances (2-way ANOVA) with post hoc Fishers LSD multiple comparisons test. Asterisks indicate significant differences in path length between hTau40$^{AT}$ mice and control group (interaction effect, genotype x day), *:p<0.05.
**(e-f)** Bar diagrams show time spent in quadrants (%) for each probe trial of (e) 10 months and (f) 16 months old hTau40$^{AT}$ mice in comparison to controls. The dotted line indicates the 25% chance level for the mice to choose the correct target region given by the presence of four quadrants (target, right, opposite, left). From probe trial 1 onwards, mice exhibit a highly significant preference for the target and avoidance for the opposite quadrant similar to control mice at 10 months of age (e), whereas the preference of the target quadrant is much less pronounced in hTau40$^{AT}$ mice at 16 months of age (f), arguing for deficits in short- and long-term memory in old transgenic animals. Bars represent mean values ± SEM. Statistics: two-tailed one sample
t-test against chance level of 25%; *:p<0.05; **:p<0.01; ***:p<0.001, ****:p<0.0001. MWM: Morris water maze, WT: wild-type animals; n: number of mice; mo: months; PT: probe trial; LTPT: long-term probe trial; opp.: opposite quadrant.

**Fig. S4: Normal motor function upon expression and accumulation of hTau40$^{AT}$ in brain and spinal cord.**
**(a)** A Rotarod test using a constant speed paradigm (16 rpm) and **(b)** an accelerating speed paradigm (4-40rpm) does not reveal any motor disabilities of hTau40$^{AT}$ in comparison to control mice at 10 and 16 months of age as scored by the latency to fall from the rotating rod (s). Bars represent group mean values ± SEM. Statistics: unpaired two-tailed Student´s t-test between transgenic mice and controls, p>0.05 for all comparisons. WT: wild-type animals; mo: months of age; n: number of mice; T1: trial 1; T2: trial 2.

**Fig. S5: No gait abnormalities due to expression and accumulation of hTau40$^{AT}$ in brain and spinal cord.**
**(a-h)** A detailed Catwalk digital foot print analysis shows no gait abnormalities of hTau40$^{AT}$ mice in comparison to controls at 12 months of age. No significant differences are detected between transgenic and control mice for parameters related to single paw or inter-limb coordination such as (a) the intensity of the paw print in arbitrary units (a.u.), expressing the mean pressure of each paw during floor contact; (b) the stride length in cm, describing the distance between two consecutive paw placements of a particular paw; (c) the swing in s, measuring the time between one step and the next for a particular paw; (d) the stance in s, indicating the amount of time a particular paw rests on the walkway; (e) the step cycle in s, exhibiting the time between two consecutive paw placements (stance

+ swing duration); (f) the duty cycle in %, showing the ratio between stance duration and step cycle duration; (g) the base of support in cm, representing the distance between the front or the hind paws; (h) the regularity index in % as a measure of inter-limb coordination defined by the percentage of normal step sequence pattern in relation to the total number of paw placements. Bars represent group mean values ± SEM. Statistics: unpaired two-tailed Student´s t-test between transgenic mice and controls, $p>0.05$ for all comparisons. WT: wild-type animals; mo: months of age; n: number of mice; FP: front paw; HP: hind paw.

**Fig. S6: Phagocytic microglia in aged hTau40$^{AT}$ mice.**
**(a-b)** Paraffin sections of 16 months old WT and hTau40$^{AT}$ mice were stained with Iba1. Note phagocytic microglia in the hilus of aged hTau40$^{AT}$ mice (b, red arrow) compared to resting microglial cells of age-matched WT mice (a). WT: wildtype; A152T: hTau40$^{AT}$ transgenic mouse strain; mo: months; Scale bar: 50µm (a-b).

**Fig. S1: Progressive Tau-phosphorylation in aging hTau40$^{AT}$ mice (5-20mo).**

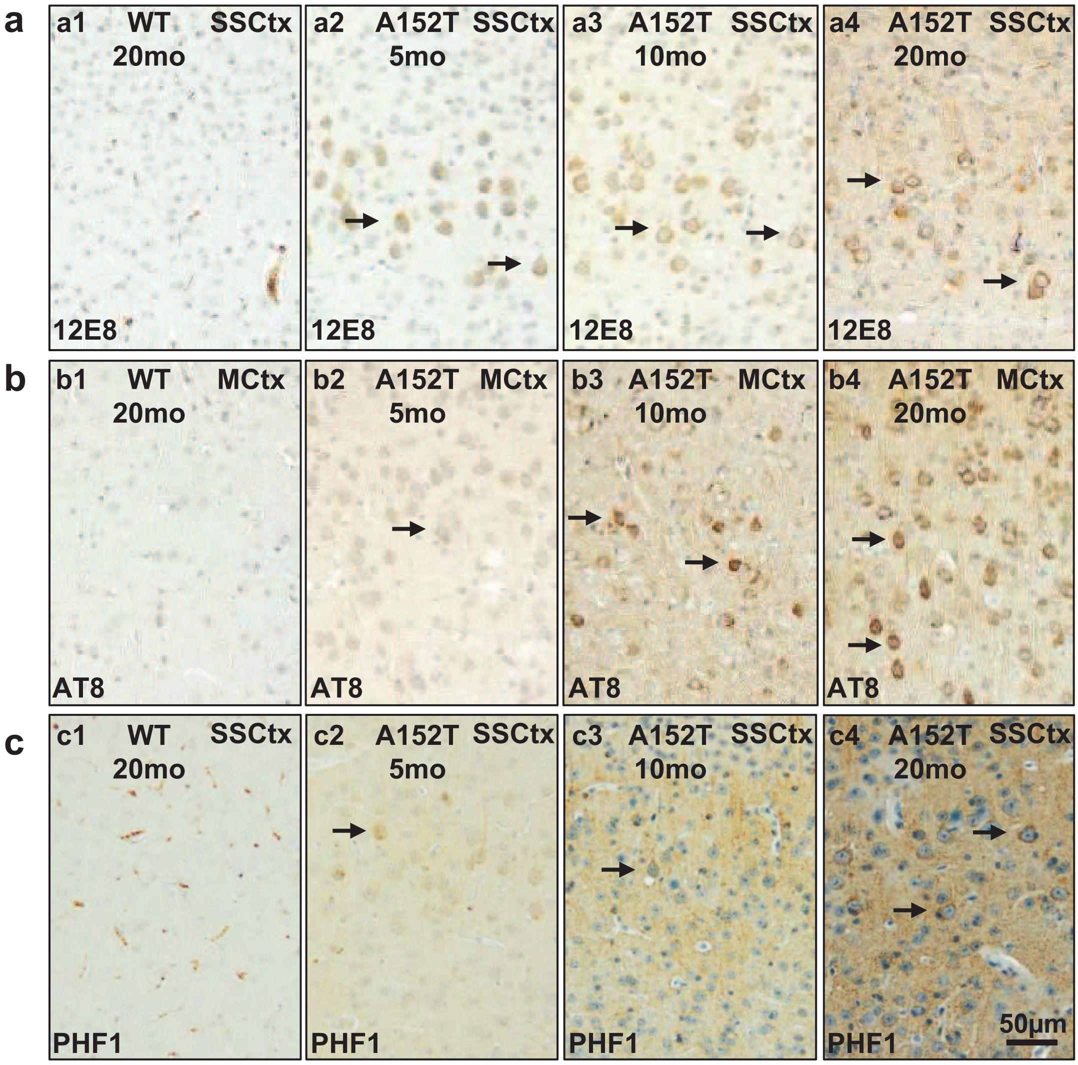

Fig. S2: No spine loss in hTau40$^{AT}$ mice at 10 months of age.

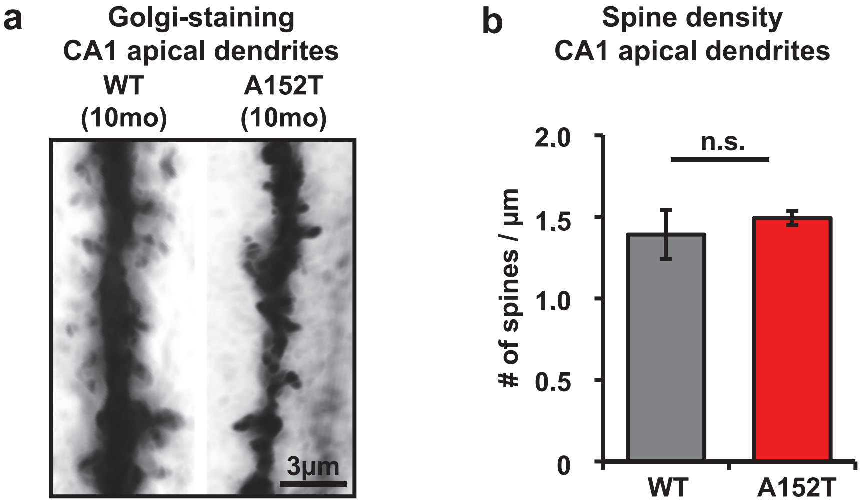

**Fig. S3: Morris water maze test shows learning and memory deficits of hTau40$^{AT}$ mice at 16 months of age.**

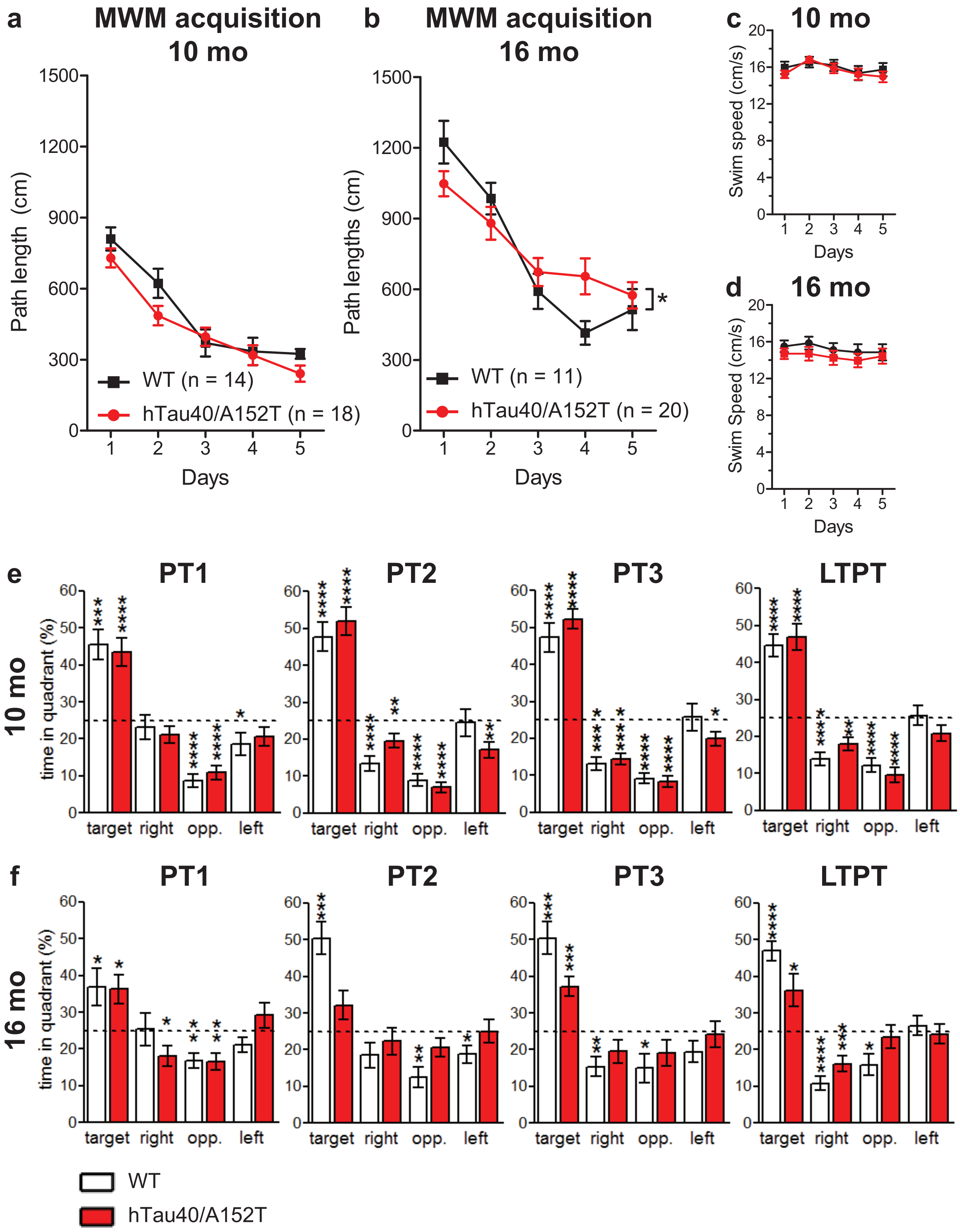

**Fig. S4: Normal motor function upon expression and accumulation of hTau40$^{AT}$ in brain and spinal cord.**

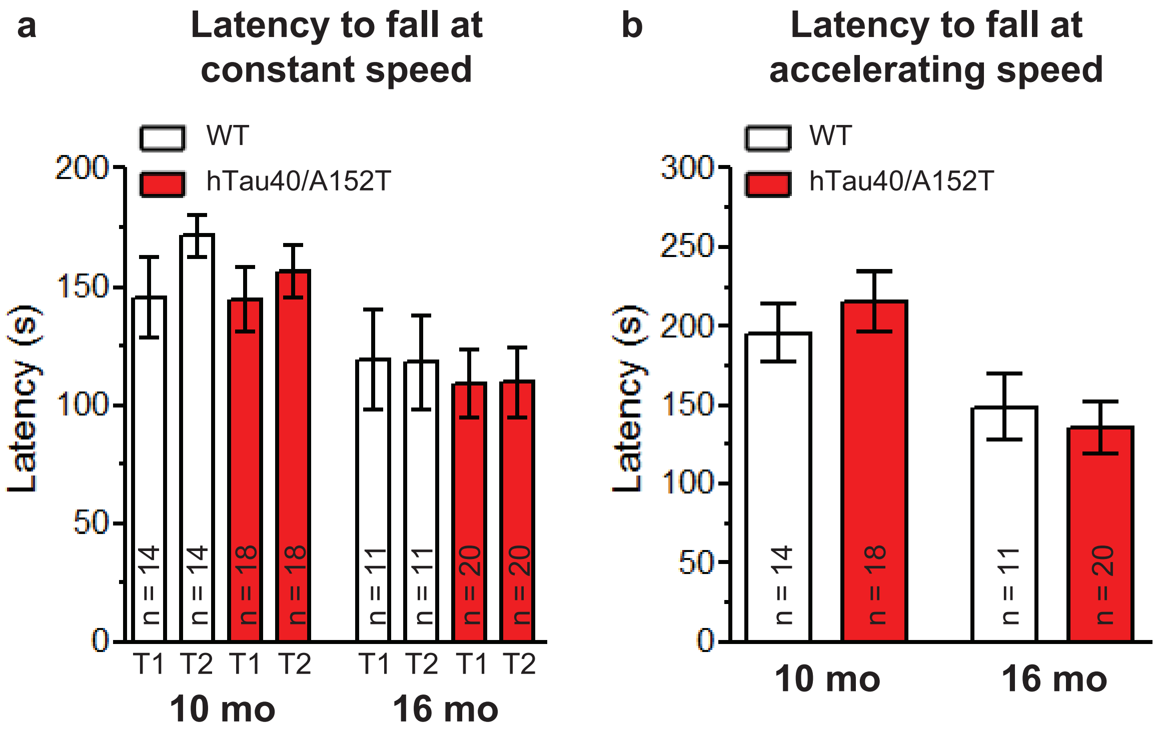

**Fig. S5: No gait abnormalities due to expression and accumulation of hTau40$^{AT}$ in brain and spinal cord.**

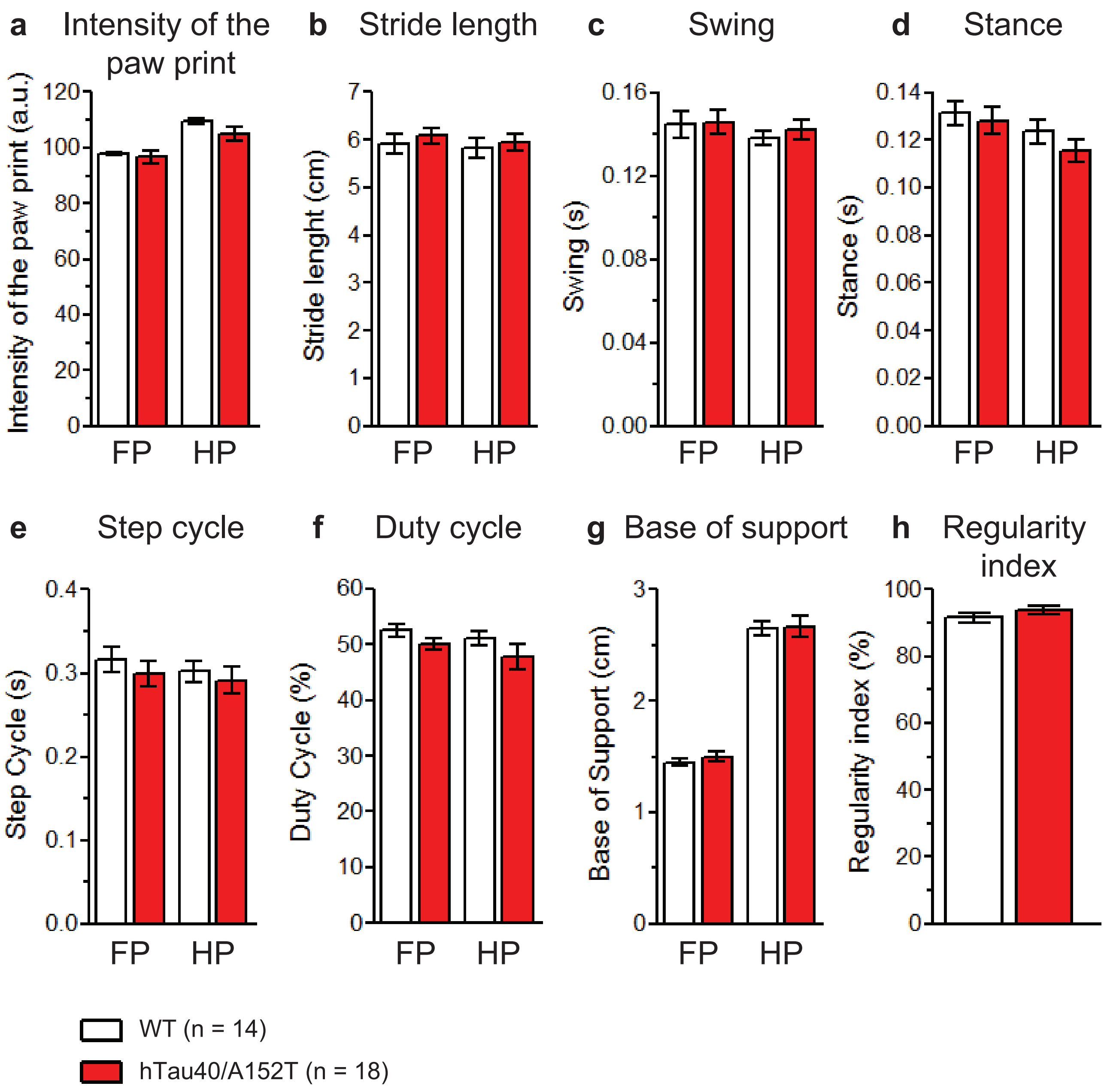

**Fig. S6: Phagocytic microglia in aged hTau40$^{AT}$ mice.**

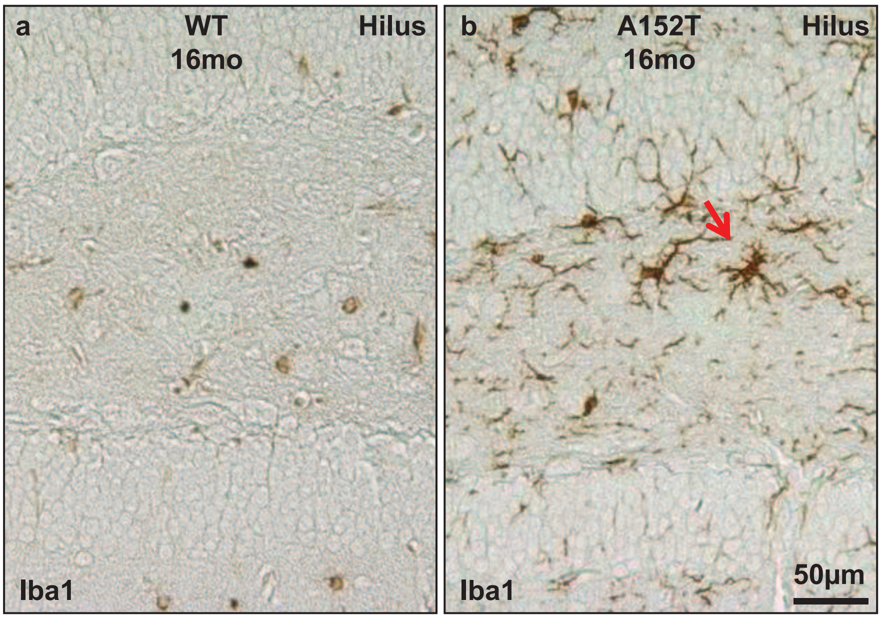

Supplement: Additional file 1: — Supplemental material and results. (PDF 5946 kb) [file 40478_2016_281_MOESM1_ESM.pdf]
